# Nanog Is the Gateway to the Pluripotent Ground State

**DOI:** 10.1016/j.cell.2009.07.039

**Published:** 2009-08-21

**Authors:** Jose Silva, Jennifer Nichols, Thorold W. Theunissen, Ge Guo, Anouk L. van Oosten, Ornella Barrandon, Jason Wray, Shinya Yamanaka, Ian Chambers, Austin Smith

**Affiliations:** 1 Wellcome Trust Centre for Stem Cell Research, University of Cambridge, Tennis Court Road, Cambridge CB2 1QR, UK; 2 Department of Biochemistry, University of Cambridge, Tennis Court Road, Cambridge CB2 1QR, UK; 3 Department of Physiology, Development, and Neuroscience, University of Cambridge, Tennis Court Road, Cambridge CB2 1QR, UK; 4 Department of Stem Cell Biology, Institute for Frontier Medical Sciences, Kyoto University, Kyoto 606-8507, Japan; 5 MRC Centre for Regenerative Medicine, Institute for Stem Cell Research, School of Biological Sciences, University of Edinburgh, King's Buildings, Edinburgh EH9 3JQ, UK

**Keywords:** STEMCELL

## Abstract

Pluripotency is generated naturally during mammalian development through formation of the epiblast, founder tissue of the embryo proper. Pluripotency can be recreated by somatic cell reprogramming. Here we present evidence that the homeodomain protein Nanog mediates acquisition of both embryonic and induced pluripotency. Production of pluripotent hybrids by cell fusion is promoted by and dependent on Nanog. In transcription factor-induced molecular reprogramming, Nanog is initially dispensable but becomes essential for dedifferentiated intermediates to transit to ground state pluripotency. In the embryo, Nanog specifically demarcates the nascent epiblast, coincident with the domain of X chromosome reprogramming. Without Nanog, pluripotency does not develop, and the inner cell mass is trapped in a pre-pluripotent, indeterminate state that is ultimately nonviable. These findings suggest that Nanog choreographs synthesis of the naive epiblast ground state in the embryo and that this function is recapitulated in the culmination of somatic cell reprogramming.

## Introduction

Pluripotency is the capacity of a single cell to generate in a flexible manner all cell lineages of the developing and adult organism. This is an essential, albeit transient, attribute of cells in embryos that undergo regulative development. After fertilization, mammalian zygotes follow a program of cleavage divisions and elaborate two extraembryonic lineages, trophoblast and hypoblast (Selwood and Johnson, 2006). This preparatory phase of development culminates in creation of the embryo founder tissue, a population of unrestricted pluripotent cells known as the epiblast (Gardner and Beddington, 1988; Nichols and Smith, 2009). The epiblast proliferates to provide the substrate for axis formation, germlayer specification, and gastrulation. Naive early epiblast cells can be immortalized in culture in the form of embryonic stem (ES) cells (Brook and Gardner, 1997; Evans and Kaufman, 1981; Martin, 1981). Pluripotent cells can also be created outside the embryo by reprogramming somatic cells, either by fusion with pre-existing pluripotent cells (Miller and Ruddle, 1976; Tada et al., 1997, 2001; Takagi et al., 1983) or, more compellingly, by transfection with regulatory transcription factors (Takahashi and Yamanaka, 2006). Information is accumulating on the molecular composition and inferred regulatory circuitry of the pluripotent state (Chen et al., 2008; Jaenisch and Young, 2008; Kim et al., 2008a). Understanding of how pluripotent cells are generated remains rudimentary, however.

Nanog is a highly divergent homeodomain-containing protein commonly accorded a central position in the transcriptional network of pluripotency (Boyer et al., 2005; Cole et al., 2008; Loh et al., 2006; Wang et al., 2006). It is essential for early embryonic development (Mitsui et al., 2003). Nanog is expressed in pluripotent embryo cells, derivative ES cells, and the developing germline of mammals and birds (Chambers et al., 2003; Lavial et al., 2007; Mitsui et al., 2003; Yamaguchi et al., 2005). Forced expression of Nanog is sufficient to drive cytokine-independent self-renewal of undifferentiated ES cells (Chambers et al., 2003).

Surprisingly, however, Nanog is not one of the canonical quartet of transcription factors employed to reprogram mouse fibroblasts (Maherali et al., 2007; Okita et al., 2007; Takahashi et al., 2007; Takahashi and Yamanaka, 2006; Wernig et al., 2007). Moreover, addition of Nanog to this quartet has not been reported to increase efficiencies. However, Nanog is expressed weakly or not at all in incompletely reprogrammed cells that fail to activate properly the endogenous pluripotent transcriptional circuitry (Silva et al., 2008; Sridharan et al., 2009; Takahashi and Yamanaka, 2006). Selection or screening for activation of endogenous *Nanog* expression facilitates isolation of fully reprogrammed induced pluripotent stem (iPS) cells that can contribute to adult chimeras and give germline transmission (Okita et al., 2007). Furthermore, in human cells Nanog does facilitate molecular reprogramming (Yu et al., 2007). It has also been shown that Nanog promotes the transfer of pluripotency after ES cell fusion (Silva et al., 2006).

*Nanog* null embryos do not develop beyond implantation (Mitsui et al., 2003). An inner cell mass (ICM) is evident in mutant blastocysts and the collapse of post-implantation development has been assumed to reflect a requirement for Nanog to maintain and expand the pluripotent epiblast (Mitsui et al., 2003). However, conditional gene deletion in ES cells revealed that Nanog is not essential for propagation of pluripotency ex vivo (Chambers et al., 2007). *Nanog* null ES cells are more prone to differentiate but can be maintained indefinitely. Moreover, they contribute extensively to somatic chimeras, presenting a major discrepancy with the embryo deletion analysis.

In this study, by clarifying the role of Nanog in generation versus maintenance of pluripotency, we seek to resolve paradoxes arising from previous findings. We compare experimental induction of pluripotency from somatic cells with natural development of pluripotency in the blastocyst.

## Results

### Nanog Dosage Is Critical for Cell Fusion-Induced Reprogramming

**Figure 1:**
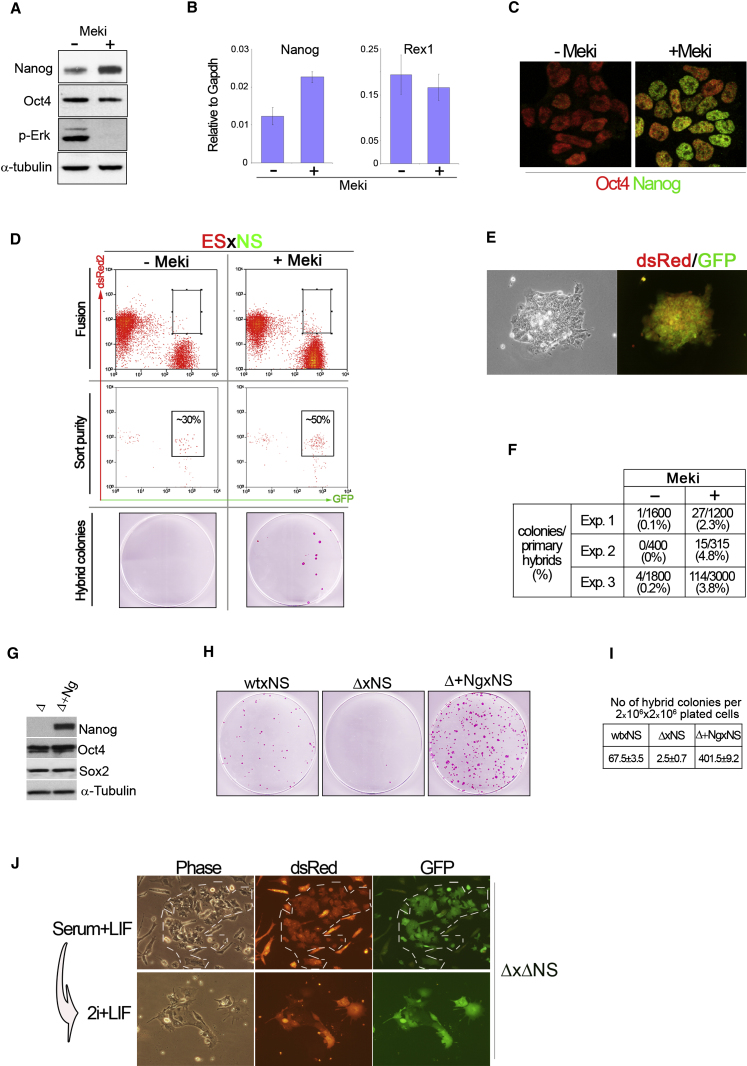
Nanog Is Critical for Transfer of Pluripotency by Cell Fusion (A) Western blot analysis for Nanog, Oct4, phospho(p)-Erk, and α-tubulin in ES cells treated with MEK inhibitor (Meki) PD184352 (3 μM). (B) Quantitative gene expression analysis for Nanog and Rex1 in Meki-treated ES cells. Error bars indicate ±1 standard deviation (SD). (C) Immunostaining for Oct4 and Nanog in control and Meki-treated ES cells. Identical settings were used for image acquisition. (D) Flow cytometric purification and analysis of primary fusion products between DsRed ES cells and GFP NS cells; alkaline phosphatase-stained plates after sorting and 9 days selection in hygromycin plus puromycin. (E) Phase contrast and fluorescence images of typical hybrid colony. Hybrids exhibit dual dsRed and GFP fluorescence from ES and NS cell genomes, respectively. (F) Quantitation of three independent fusions. Scores are normalized for purity determined by flow cytometry analysis. MEK inhibitor was PD184352 in the first two experiments and PD0325901 in the third. (G) Western blot analysis for Nanog, Oct4, and Sox2 in *Nanog*^−/−^ and *Nanog*^−/−^ ES cells transfected with a Nanog transgene (*Ng)*. (H) Plates containing hybrid colonies stained for alkaline phosphatase from fusions (x) of ES cells (WT, Δ, and Δ*Ng*) with NS cells. Cells were grown under puromycin selection for reactivation of the Oct4 reporter transgene. (I) Mean number of hybrid colonies per plate from two replicate experiments. (J) Images of culture generated from cell fusion between GFP-expressing ΔES cells and GFP/dsRed-expressing ΔNS cells. Upper panels show a hybrid colony (dashed line) after 2 weeks under puromycin and hygromycin selection. Approximately 10 of these colonies were observed per plate. Lower panels show parallel culture switched to 2i/LIF plus puromycin for 4 days to select specifically for pluripotent hybrids (bottom panels).

Transgenic expression of Nanog promotes formation of pluripotent hybrids after fusion of ES cells with somatic cells (Silva et al., 2006). We investigated whether upregulation of endogenous *Nanog* may have a similar effect. Exposure of ES cells to 3 μM MEK inhibitor (PD184352 or PD0325901) (Ying et al., 2008) in the presence of serum and leukemia inhibitory factor (LIF) results in increased expression of Nanog without altering levels of Oct4 (Figures 1A and 1B). Rex1, a sensitive indicator of undifferentiated ES cell status (Toyooka et al., 2008), is also unchanged suggesting that the increase in Nanog is not secondary to reduced differentiation. Nanog has been shown to fluctuate in ES cells cultured in serum and LIF (Chambers et al., 2007). MEK inhibition increases the fraction of Nanog-positive cells to over 90% and also increases the mean and maximum levels of expression (Figure 1C and Figure S1 available online). We treated ES cells with 3 μM MEK inhibitor for 3 days prior to polyethylene glycol (PEG) mediated fusion with brain-derived neural stem (NS) cells. The NS cells constitutively express tauGFP and puromycin resistance whereas the ES cells express the dsRed2 and hygromycin resistance, enabling detection and selection of hybrids (Silva et al., 2006). Fused cells were purified by flow cytometry 24 hr after PEG treatment, quantitated (Figure 1D), and plated in complete ES cell medium. MEK inhibitor was maintained for 72 hr after sorting, then withdrawn. Puromycin plus hygromycin selection was then applied. Macroscopic colonies of typical ES cell morphology emerged after 5–6 days under selection. All of these expressed GFP and dsRed2 (Figure 1E). Plates were fixed on day 12 and stained for alkaline phosphatase, a marker of ES cells (Figure 1D). MEK inhibitor-treated cultures yielded a greater than 40-fold increase in undifferentiated hybrid colonies, normalized to the number of fused cells plated to eliminate variation due to differences in fusion efficiency (Figure 1F). This dramatic effect of MEK inhibition is likely to be mediated at least in part via upregulation of Nanog since endogenous Nanog is normally limiting for transfer of the pluripotent state (Silva et al., 2006).

We then exploited the availability of *Nanog* null (Δ) ES cells (Chambers et al., 2007) to evaluate whether Nanog may be necessary to produce pluripotent hybrids. As controls we employed both parental ES cells and *Nanog*Δ cells with restored Nanog expression from a constitutive transgene (Δ+*Ng*) (Figure 1G). Fusion of parental ES cells with Oct4-GiP NS cells generated around 70 colonies per plate (Figures 1H and 1I). Δ+*Ng* ES cells yielded increased numbers of hybrid colonies, >400 per plate, consistent with the enhancing effect of overexpression of Nanog (Silva et al., 2006). In contrast, fusions using *Nanog*Δ ES cells yielded only 2–3 hybrid colonies in identical conditions (Figures 1H and 1I). This 25-fold reduction in hybrid yield indicates that Nanog expression is a critical determinant for the efficiency of reprogramming. Furthermore the occasional hybrid colonies recovered from *Nanog*ΔES×NS cell fusions expressed Nanog from the NS cell genome (Figure S2). Activation of Nanog after fusion could compensate for absence from the ES cell partner. We therefore fused *Nanog*Δ ES cells with *Nanog*Δ NS cells (Figure S3). We introduced into the *Nanog*Δ NS cells a dual puromycin resistance and dsRed expression construct. After fusion, 4 × 10^6^ cells were plated per 10 cm dish and placed under selection the following day. A few hybrid colonies, approximately 10 per plate, were evident after 2 weeks, identified by combined red and green fluorescence. These cells did not have typical compact ES cell colony morphology, however (Figure 1J). To clarify their status we transferred them to the selective ground state culture combination of MEK inhibitor and GSK3 inhibitor (2i) plus LIF (Ying et al., 2008). NS cells cannot survive in 2i and puromycin was maintained to eliminate any persisting unfused ES cells. All cells died or differentiated within days. In contrast hybrids generated between null ES cells and null NS cells expressing a *Nanog* transgene expanded readily without differentiation. Nanog is not required by established pluripotent cells in 2i/LIF because *Nanog* null ES cell lines can readily be propagated (Figure S4). We conclude that if Nanog is absent from both fusion partners, hybrid cells do not attain pluripotency.

### Nanog Is Necessary for Molecular Reprogramming to Progress beyond Dedifferentiation to Pluripotency

**Figure 2:**
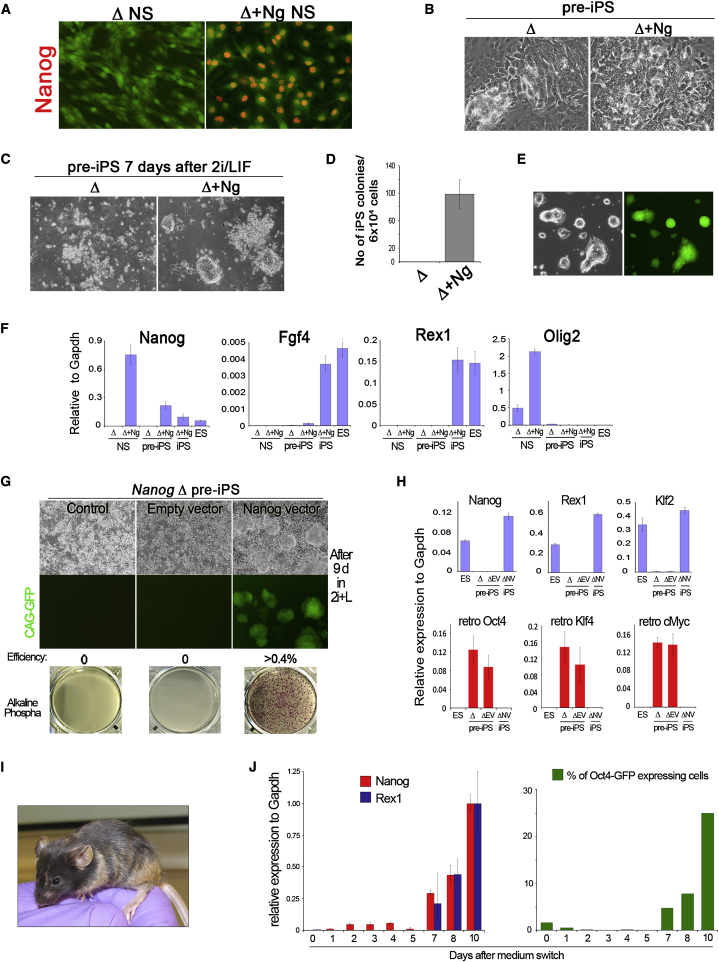
Nanog Is Necessary for Full Reprogramming to Induced Pluripotency (A) Immunostaining for Nanog in Δ+*Ng* NS cells. (B) Morphology of Δ and Δ+*Ng* NS cells 5 days after transduction with c-Myc, Oct4, and Klf4. (C) *Nanog*Δ and Δ+*Ng* partially reprogrammed cells 7 days after switching to 2i medium. (D) Number of iPS cell colonies per 6 × 10^4^ plated pre-iPS cells. Counts were performed 10 days after 2i medium switch. Selection in G418 for *Nanog* promoter activity was applied 4 days prior to counts. Error bars indicate ±1 SD. (E) Passaged Δ+Ng iPS cells cultured in 2i. (F) Quantitative gene expression analysis for Nanog, Fgf4, Rex1, and Olig2 in Δ and Δ+*Ng* NS cells, Δ and Δ+*Ng* pre-iPS cells, and Δ+*Ng* iPS cells. Error bars indicate ±1 SD. (G) Response of Δ, Δ empty vector (EV), and Δ Nanog vector (NV) pre-iPS cells to culture in 2i/LIF for 9 days. For each line 4 × 10^5^ cells were plated on feeders in duplicate wells. Approximately 1400 ΔNV iPS colonies were obtained per well, corresponding to an efficiency of 0.4%. (H) Quantitative gene expression analysis for Nanog, endogenous Klf2, and Rex1 and for retroviral (retro) Oct4, Klf4, and c-Myc in ΔNV iPS cells and Δ and ΔEV pre-iPS cells. Error bars indicate ±1 SD. (I) Coat color chimera obtained by blastocyst injection of Nanog vector Δ iPS cells after Cre excision of the *Nanog* transgene. (J) Time course of Nanog and Rex1 mRNA expression (left panel) and emergence of Oct4-GFP-positive cells (right panel) following transfer of MEF-derived pre-iPS cells to 2i/LIF. Flow cytometry scatter plots are provided in Figure S7. Error bars indicate the range of fold change relative to the sample with highest expression.

The hybrid results led us to hypothesize that Nanog may be dispensable in the initial stages of reprogramming the somatic cell genome but essential for instating pluripotency. We therefore examined requirements for Nanog during molecular reprogramming (Takahashi and Yamanaka, 2006). NS cells undergo rapid reprogramming after transfection with Oct4, Klf4, and c-Myc, without requirement for exogenous Sox2 (Kim et al., 2008b; Silva et al., 2008). As a control population we introduced a constitutive *Nanog* transgene (*Ng*) into *Nanog*Δ NS cells (Figure 2A). Oct4, Klf4, and c-*myc* were transduced into *Nanog*Δ and Δ+*Ng* NS cells. After 3 days, cells were transferred into medium containing serum and LIF. Two days later the plates were near-confluent with compact proliferating cells that had lost the bipolar morphology of NS cells and morphologically resembled undifferentiated ES cells (Figure 2B) (Silva et al., 2008). These cells downregulated the NS cell marker Olig2 and acquired the intercellular adhesion molecule Ecadherin (Figure S5B). They expressed ES cell markers SSEA-1 and alkaline phosphatase (Figures S5A and S5C). However, endogenous core pluripotency factors were not robustly expressed (Figure 2F). There were no discernible differences between transduced *Nanog*Δ and Δ+*Ng* cells. These observations suggest that Nanog is not required to produce a dedifferentiated partially reprogrammed state.

Incompletely reprogrammed cells may be promoted to pluripotency by exposure to 2i/LIF (Silva et al., 2008). This allowed us to test whether partially reprogrammed *Nanog*Δ cells could transit to pluripotency. *Nanog*Δ cells transduced with the three factors were passaged once on feeders in the presence of serum and LIF, then switched to 2i/LIF. All cells died within 7 days (Figure 2C). No colonies formed from multiple wells plated (Figures 2C and 2D). In marked contrast Δ+*Ng* cells treated in the same way generated around 100 colonies per plate of 6 × 10^4^ cells, corresponding to a conversion frequency of 0.16% ± 0.02% (Figures 2C and 2D). These colonies were expanded in 2i/LIF and stable lines generated (Figure 2E). Resistance to G418 and hygromycin indicated activation of the endogenous targeted *Nanog* alleles. They expressed Fgf4 and Rex1 unlike the partially reprogrammed cells (Figure 2F).

We deleted the floxed *Nanog* transgene after conversion in 2i/LIF (Figure S6A) by Cre recombination (Chambers et al., 2007). The deletion is conveniently monitored by gain of dsRed expression. DsRed-positive iPS cells continued to proliferate with undifferentiated morphology and retention of mRNA markers of pluripotency (Figure S6B). To confirm ground state identity and developmental potential, we examined ability to colonize the mouse embryo after morula aggregation. Chimeric fetuses analyzed at mid-gestation exhibited widespread contribution of GFP-positive cells (Figure S6D). Therefore, in line with results in ES cells (Chambers et al., 2007), Nanog is no longer necessary in iPS cells once pluripotency has been attained by reprogramming. We conclude that Nanog is specifically required for partially reprogrammed pre-iPS cells to reach ground state pluripotency.

### Nanog Enables pre-iPS Cells to Acquire Ground State Pluripotency

We then investigated whether partially reprogrammed *Nanog*Δ cells that appear incapable of progression to the ground state are indeed pre-iPS cells. To do this we restored Nanog expression using *PiggyBac* transposition (Wang et al., 2008) to introduce a floxed *Nanog* transgene into the partially reprogrammed cells. Stable transfectants were obtained after hygromycin selection. Cells were then transferred into 2i/LIF as above. In contrast to nontransfected and empty vector transfected cells that died, *PB-Nanog* transfectants produced over 1400 colonies per well (Figure 2G). This corresponds to a conversion efficiency of more than 0.4%. These cells are G418 resistant, indicating activation of the endogenous targeted *Nanog* locus. qRT-PCR analysis revealed silencing of retroviral expression and upregulation of ground state pluripotency markers Rex1 and Klf2 to levels comparable to those found in ES cells (Figure 2H). After tamoxifen-induced Cre excision of the Nanog transgene these cells gave liveborn coat color chimeras (Figure 2I).

We then investigated the timing of gene activation in pre-iPS cells after the switch to 2i/LIF. We exploited a clonal line of pre-iPS cells derived from mouse embryo fibroblasts. These cells do not express Nanog or Rex1 (Figure S7), and when maintained in the presence of serum they do not spontaneously acquire pluripotency even under selection for Oct4 promoter activation. However, they convert to germline-competent pluripotency at high efficiency after transfer to 2i/LIF. Conversion takes place over a period of 10 days (Silva et al., 2008). Strikingly, we found that Nanog mRNA is only significantly upregulated from day 7. This timing is coincident with the upregulation of endogenous pluripotency markers and of an *Oct4-GFP* transgene reporter (Figures 2J and S7). These data indicate that Nanog is not the immediate target of 2i in pre-iPS cells. Consistent with this interpretation, transgenic expression of Nanog in pre-iPS cells is not sufficient to enable efficient escape from the pre-iPS cell state without exposure to 2i/LIF. Together, these data indicate that Nanog acts at the final stage of the molecular reprogramming process to instate pluripotency.

### Transient Nanog Mediates Reprogramming of EpiSCs

**Figure 3:**
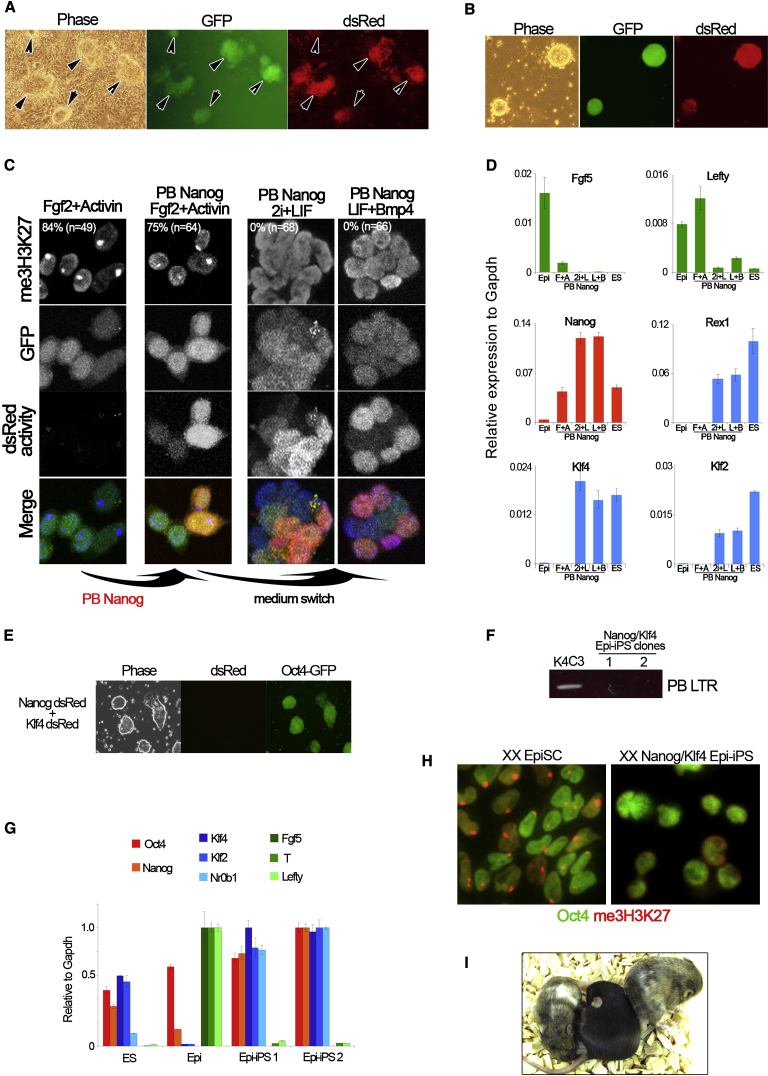
Stable Transfection of EpiSCs with Nanog Enables Conversion to iPS Cells (A) Representative image of EpiSCs stably transfected with PB-Nanog-dsRed then transferred for 4 days to 2i/LIF. Arrowheads indicate groups of cells expressing Oct-GFP and the PB-Nanog-dsRed transgene. (B) PB-Nanog-dsRed EpiSC transfectants cultured for 7 days in LIF+Bmp4, under puromycin selection from day 4. (C) Immunostaining for me3H3K27 and fluorescence imaging of EpiSCs, PB-Nanog-dsRed (PBNanog) EpiSCs, and PBNanog transfectants after 1 passage in 2i+LIF or LIF+Bmp4. (D) Quantitative gene expression analysis in EpiSCs (Epi), PBNanog EpiSCs in Fgf2+Activin (F+A), and passage 1 PB-Nanog transfectants in 2i+LIF (2i+L) or LIF+Bmp4 (L+B), compared with ES cell sample. Error bars indicate ±1 SD. (E) Expanded clone of Epi-iPS cells generated by transient transfection of EpiSCs with PB-Nanog-dsRed plus PB-Klf4-dsRed. (F) Genomic PCR analysis for Piggybac LTR in Epi-iPS cells generated by transient transfection. K4C3 is an iPS cell line generated by stable transfection of PB-Klf4 and provides a positive control. (G) Quantitative mRNA expression analysis of Epi-iPS cells produced by transient transfection. Error bars indicate the range of fold change relative to the sample with highest expression. (H) Immunostaining for me3H3K27 shows nuclear foci corresponding to X_i_ in EpiSCs and absence in derivative Epi-iPS cells generated by transient transfection. (I) Coat color chimeras obtained from Epi-iPS cells generated by transient transfection of EpiSCs with PB-Nanog-dsRed plus PB-Klf4-dsRed.

EpiSCs are a cell type in which many components of pluripotent circuitry are naturally present. They are derived from the epithelialized epiblast of post-implantation embryos (Brons et al., 2007; Tesar et al., 2007) or by differentiation of ES cells (Guo et al., 2009). EpiSCs are distinct from ES cells in gene expression, growth factor dependency, inactivation of the X chromosome (in XX cells), and inability to contribute to blastocyst chimeras. This is a transcriptionally and epigenetically differentiated cell state compared with naive epiblast or ES cells (Nichols and Smith, 2009) that does not revert, even in 2i/LIF. However, EpiSCs can be reprogrammed to ground state pluripotency by stable transfection with Klf4 and culture in 2i/LIF. This occurs with an efficiency of 0.1%–1%, similar to somatic cell reprogramming (Guo et al., 2009). EpiSCs express Nanog but at lower levels than ES cells. We therefore tested whether elevated expression of Nanog may mediate conversion to the ground state. We used EpiSCs derived from an E5.5 female embryo carrying the Oct4-GiP reporter. Nanog stable transfectants were generated by lipofection with a *Piggybac* vector containing independent promoters directing expression of Nanog and of dsRed reporter linked to the hygromycin resistance gene (PBNanogdsRed). Transfectants were expanded in EpiSC culture conditions of Fgf2 and activin under hygromycin selection. Under these conditions there is no upregulation of ground state markers. Upon transfer to 2i/LIF, which eliminates parental EpiSCs or control transfectants (Guo et al., 2009), multiple undifferentiated colonies form that express Oct4-GFP (Figure 3A). Colonies were obtained at an average of 200–2000 per well of 2 × 10^4^ cells, or 1%–10%. These cells could be stably propagated and exhibited upregulation of ES cell markers (Figure 3D). This frequency of conversion is 10-fold higher than for Klf4 transfectant in side-by-side experiments. Klf4-mediated conversion is dependent on treatment with 2i, which may act in part by inducing Nanog. We asked whether Nanog can mediate reprogramming of EpiSCs without 2i. Nanog-transfected EpiSCs transferred from Fgf2/activin culture to medium supplemented with LIF+BMP4 produced numerous undifferentiated Oct-GFP-positive colonies (Figures 3B and 3C) that could be stably expanded and showed the transcriptional marker profile of ground state pluripotency, including upregulation of Klf4 (Figure 3D). No iPS cell colonies were obtained from parallel cultures of Klf4 transfectants without 2i.

The Nanog-generated Epi-iPS cell colonies invariably expressed dsRed (Figure 3C) indicating integration of the transgene. Transient transfection of the PB-Nanog construct without PBase did not yield any Epi-iPS cells. We then tested transient cotransfection of Nanog with Klf4. Transfected cells were transferred into 2i/LIF after 48 hr. Epi-iPS cell colonies emerged over the following 7 days at a frequency of 2–6 colonies per well of 2 × 10^4^ starting EpiSCs. Several of these colonies were picked and expanded. They showed uniform expression of Oct4-GFP but no dsRed (Figure 3E). Integration of PB vectors was undetectable by genomic PCR (Figure 3F). qRT-PCR analysis confirmed transcriptional resetting with upregulation of ES cell markers Nr0b1, Klf4, and Klf2 and downregulation of EpiSC markers Fgf5, Brachyury, and Lefty (Figure 3G). Immunostaining for the silencing mark me3H3K27 revealed that the nuclear focus corresponding to the inactive X chromosome was erased in the Epi-iPS cells (Figure 3H). The definitive test of ground state pluripotency is ability to colonize the developing embryo and contribute substantially to term chimeras. Two clones of Nanog/Klf4 Epi-iPS cells were tested by morula aggregation and both colonized the egg cylinder at high frequency (Figure S8), a property never exhibited by EpiSCs (Guo et al., 2009). After blastocyst injection of one of these clones, healthy adult mice were obtained with extensive coat color chimerism (Figure 3I).

These findings confirm that in the presence of other key pluripotency factors, Nanog can orchestrate transition to the ground state, mediating both transcriptional and epigenetic reprogramming. Intriguingly, as previously noted for ES cell self-renewal (Chambers and Smith, 2004) and hybrid reprogramming (Silva et al., 2006), endogenous expression of Nanog is limiting, indicating dosage sensitivity in Nanog action.

### Nanog Protein Localizes Specifically to Nascent Epiblast

**Figure 4:**
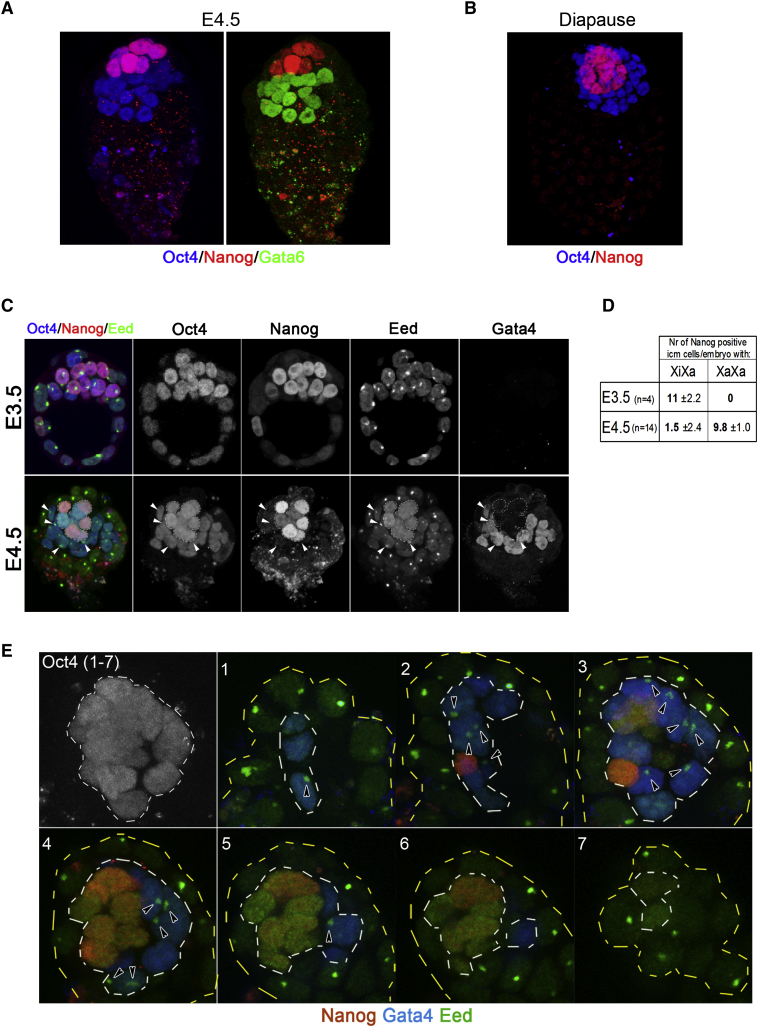
Nanog Expression Demarcates the Epiblast and Is Coincident with the Domain of X_i_ Reactivation (A) Immunostaining for Oct4, Nanog, and Gata6 in E4.5 embryos. (B) Immunostaining for Oct4 and Nanog in diapause embryos. (C) Single optical sections through E3.5 and E4.5 embryos immunostained for Oct4, Nanog, Eed, and Gata4. White arrows point to cells in E4.5 embryos stained with Oct4 and displaying Eed foci (inactive X chromosome). (D) Eed status in Nanog-positive ICM cells of blastocysts with Eed foci in trophoblast nuclei. Nanog-positive cells were scored as X_i_X_a_ when an Eed nuclear body was present and X_a_X_a_ when this was absent. Counts show average number (Nr) of cells per embryo with the indicated phenotype. (E) Confocal sections through ICM region of an E4.5 blastocyst immunostained for Eed, Oct4, Nanog, and Gata4. Oct4 staining marks the ICM (white dashed line), whereas Eed staining alone represents trophectoderm nuclei (between yellow and white dashed line). Only Nanog-stained nuclei lack the Eed nuclear body.

The preceding evidence that Nanog is crucial for instating pluripotency during somatic cell reprogramming prompted re-examination of its role in embryogenesis. Nanog is essential for development after implantation (Mitsui et al., 2003). This could be because it serves to maintain the epiblast or because it is required to make a pluripotent epiblast. Nanog is present from the late morula throughout formation and expansion of the ICM (Chambers et al., 2003; Dietrich and Hiiragi, 2007). The epiblast emerges through partition of the ICM in the mature mouse blastocyst between embryonic day (E) 3.5 and E4.5 (Gardner, 1983). Nanog mRNA is downregulated shortly thereafter (Chambers et al., 2003). We analyzed distribution of Nanog protein at these stages (Figures 4 and S9).

We compared localization of Nanog with Oct4, generally considered a pluripotency marker but also transiently expressed during hypoblast formation (Palmieri et al., 1994). We contrasted Nanog distribution with Gata6 and Gata4, specification factors for the hypoblast (Chazaud et al., 2006). At E3.5 Nanog was largely confined to the ICM (Figures 4C and S9) and present in most, though not all, of these cells, as previously described (Chazaud et al., 2006; Dietrich and Hiiragi, 2007). Oct4 was strongly expressed throughout the ICM and also detectable in some trophoblast cells. By E4.5 Nanog immunostaining was confined to a subset of ICM cells overlaid on one surface by trophoblast and on the other by hypoblast (Figure 4A). Oct4 was expressed more broadly and was present in almost all ICM cells (Figure 4). Gata4 and Gata6 were restricted to ICM cells lining or close to the blastocoel, corresponding to the location of the hypoblast (Figures 4A and 4C). Many of those cells coexpressed Oct4. In contrast, we did not observe at E4.5 any instance of a cell that coexpressed Nanog with either Gata4 or Gata6.

Mouse embryo development can be arrested and synchronized at the mature blastocyst stage if implantation is prevented by ongoing lactation or by experimental deprivation of estrogen (Nichols et al., 2001). This condition of diapause is a favored stage for the derivation of ES cells (Evans and Kaufman, 1981). Oct4 is expressed in all ICM cells in diapause. In contrast Nanog is present only in the interior ICM cells and is absent from the hypoblast layer (Figure 4B). These findings establish that Nanog uniquely marks the epiblast in the mature blastocyst and is mutually exclusive with expression of the Gata factors, whereas Oct4 is ubiquitous throughout the ICM.

### Expression of Nanog Defines the Domain of X Chromosome Reactivation

**Figure 5:**
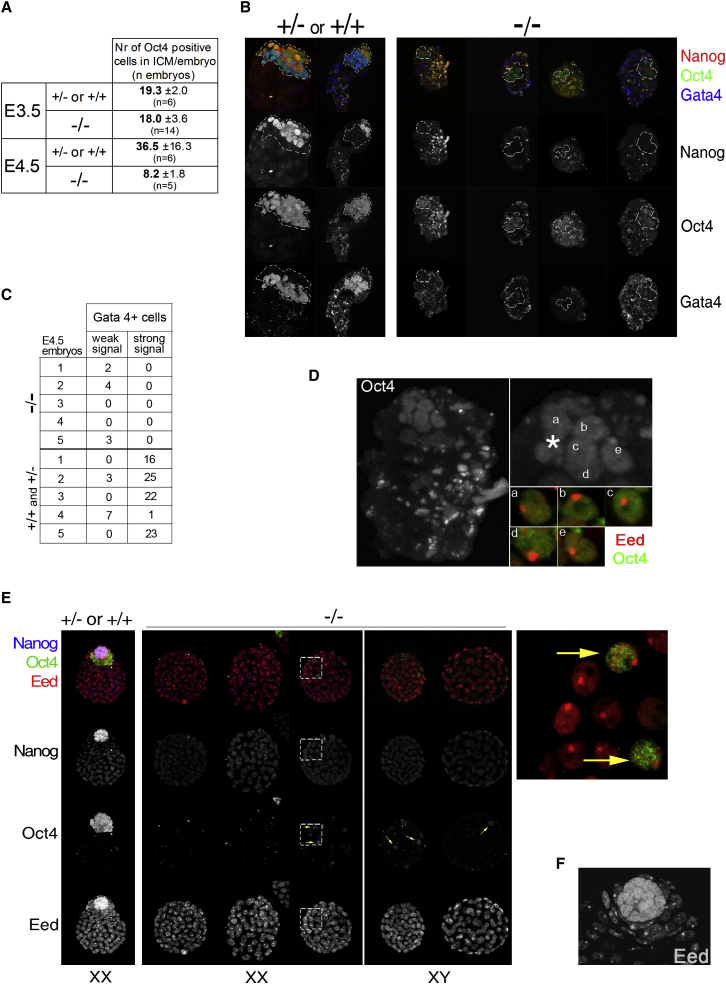
Phenotypic Analysis of *Nanog* Null Blastocysts (A) Counts of the total number of Oct4-stained internal cells in blastocysts from *Nanog*^+/−^ intercrosses determined by confocal analysis. (B) Nanog, Oct4, and Gata4 immunostaining of E4.5 blastocysts. (C) Counts for total number of Gata4-positive cells with weak or strong signal in E4.5 embryos. (D) Nanog-negative E4.5 blastocyst stained for Oct4 and Eed. Asterisk marks shrunken nucleus of dying cell. Insets show Eed nuclear bodies in Oct4 stained cells. (E and F) Nanog, Oct4, and Eed immunostaining of diapause blastocysts. Gender of the blastocyst inferred from Eed staining is indicated at the bottom of each panel. (E) Arrows indicate Oct4-positive cells in Nanog-negative blastocysts. Right panel shows zoomed image from a mutant diapause embryo (dashed square) stained for Oct4 and Eed. This shows two cells expressing Oct4 and exhibiting the Eed mark of an inactive X chromosome. (F) ICM region of a Nanog-positive diapause embryo showing absence of Eed foci in Nanog-positive cells.

In female mouse embryos the paternal X chromosome is silenced during early cleavage (Okamoto et al., 2004). The inactive X (X_i_) is then reactivated in the ICM (Mak et al., 2004; Okamoto et al., 2004). Reactivation is critical to allow for random X inactivation in the embryo proper and may be considered an essential attribute of ground state pluripotency in females. Indeed a distinctive feature of female ES cells is that both X chromosomes are active (Martin, 1981; Rastan and Robertson, 1985). Furthermore, ES cells have the capacity to reactivate a silenced somatic X chromosome during cell fusion-induced reprogramming (Silva et al., 2006; Tada et al., 2001; Takagi et al., 1983). We therefore analyzed the relationship between Nanog expression and reactivation of X_i_ in the ICM. We used immunostaining for Eed, a component of the PRC2 polycomb group complex, to detect epigenetic marking indicative of X_i_ (Silva et al., 2003). We compared the presence or absence of an Eed nuclear body with Nanog expression. All ICM cells of XX E3.5 blastocysts exhibited a prominent Eed focus (Figures 4C and 4D), confirming that X_i_ is maintained in the early blastocyst (Mak et al., 2004). Therefore coexpression of Nanog, Oct4, and Sox2, at least at the levels present at E3.5, is not sufficient for X chromosome reactivation. At E4.5, however, around half of the Oct4-positive ICM cells in XX blastocysts had lost the Eed nuclear body that was readily detectable in neighboring trophoblast cells (Figures 4C and 4E). Absence of the Eed focus correlated precisely with the presence of Nanog (Figures 4C and 4E and Movie S1). ICM cells retaining X_i_ did not have Nanog but expressed Gata4 (Figures 4C and 4E), indicative of hypoblast differentiation (Chazaud et al., 2006). Similarly in diapause blastocysts an Eed nuclear body was evident in all cells of the hypoblast and trophoblast but absent from Nanog-positive epiblast cells (Figure 5F). From all embryos examined, every cell without an Eed nuclear body also expressed Nanog. These observations indicate that X chromosome silencing is erased in only a subset of the ICM.

We conclude that Nanog expression at E4.5 defines the embryo founder compartment and is coincident with the domain of X chromosome reactivation. Nanog-negative ICM cells still express Oct4 but are Gata6/Gata4 positive. These hypoblast cells do not erase X chromosome silencing. Thus paternal X chromosome inactivation in the yolk sac endoderm does not entail a second cycle of imprinted inactivation but is inherited from early cleavage as occurs in the trophoblast.

### *Nanog* Null ICMs Fail to Reactivate X_i_ and Do Not Generate Pluripotent Cells

We then analyzed ICM status in *Nanog*^−/−^ blastocysts (Mitsui et al., 2003). At E3.5 Nanog protein was undetectable in one quarter of intercross blastocysts, but these were not distinguishable in morphology, size, or ICM cell number (Figures 5A and S9). In contrast, an overt phenotype was evident in embryos harvested at E4.5. Of 57 E4.5 intercross blastocysts, 12 lacked detectable Nanog protein. The Nanog-negative specimens contained substantially fewer ICM cells, and these showed reduced intensity of Oct4 staining (Figures 5A and 5B). Strong Gata4 staining was lacking, though faint immunoreactivity was apparent in a few cells (Figures 5B and 5C). In five Nanog-negative blastocysts with Eed foci in trophoblast nuclei, the nuclear body was also prominent in all intact ICM nuclei (Figure 5D). Persistence of X_i_ in Oct4-positive ICM cells in mutant embryos at E4.5 is consistent with a putative direct role for Nanog in X chromosome reactivation (Navarro et al., 2008). It also points to a failure of null ICM cells to develop into epiblast.

To clarify the cellular basis of the phenotype of Nanog-deficient blastocysts, we examined diapause blastocysts. Diapause effectively extends the preimplantation period and allows the possibility for retarded cells to catch up in development. Eight of thirty-one intercross diapause blastocysts lacked Nanog-positive cells. None of these contained an overt ICM compartment. Four were completely Oct4 negative, while the others contained a few Oct4-stained cells dispersed around the trophectoderm layer (Figure 5E). In Nanog-negative diapause embryos that showed Eed foci, this was present in every nucleus including those staining for Oct4 (Figure 5E), indicating that X reactivation had not occurred. These observations indicate that the reduced size of the ICM at E4.5 and the absence of hypoblast are not due to retarded growth or differentation but are a result of developmental failure. We surmised that Nanog may be required to achieve a pluripotent ground state in the early embryo.

**Figure 6:**
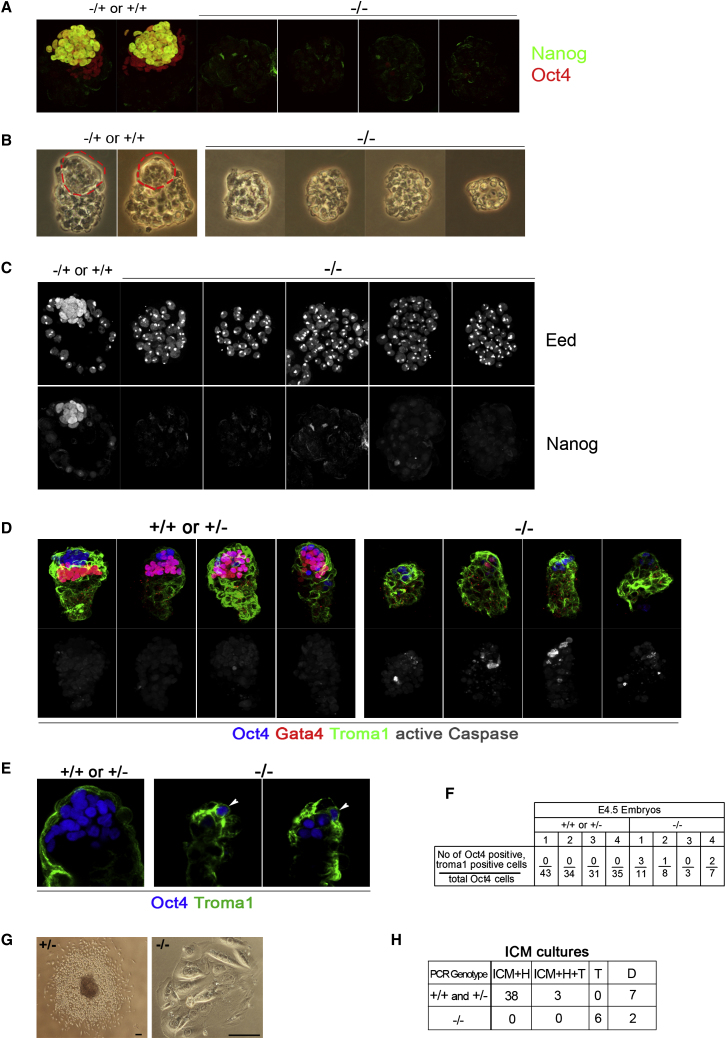
*Nanog* Null Blastocysts Do Not Generate Pluripotent ICM Cells (A–C) Blastocysts were harvested at E3.5 from *Nanog*^+/−^ intercrosses and cultured in suspension in 2i/LIF for 2 days. (A) Immunofluorescence staining for Nanog and Oct4. (B) Phase contrast images. Dashes outline ICMs. (C) Eed and Nanog immunostaining. (D) Oct4, Gata4, Troma1, and active caspase immunostaining of freshly harvested E4.5 blastocysts. (E) Single optical sections of E4.5 blastocysts stained for Oct4 and Troma1. Arrowhead points to double-positive cells apparently located in the surface trophoblast. (F) Counts for number of Oct4-positive Troma1-positive cells. (G) Examples of 8 day cultures of ICMs isolated from *Nanog*^+/−^ intercross E3.5 blastocysts. Left panel: culture from heterozygous ICM showing persistent ICM and outgrowing hypoblast (H) cells. Right panel: trophoblast (T) sheet differentiated from null ICM. (H) Table summarizing cultures of outgrowths from *Nanog*^+/−^ intercross E3.5–E3.75 ICMs. D: degenerated embryos.

To test further whether pluripotent cells could be produced in the absence of Nanog we examined responsiveness to 2i. Culture of early embryos in 2i maximizes expansion of the epiblast (Nichols et al., 2009a) and facilitates subsequent derivation of ES cells, including from otherwise recalcitrant strains (Nichols et al., 2009b; Ying et al., 2008). Early blastocysts cultured in 2i or 2i/LIF for 48 hr develop expanded Oct4-positive ICMs with a large component of Nanog-expressing cells along with Gata4-positive hypoblast (Figure 6A). *Nanog* null ES cells expand more efficiently in 2i or 2i/LIF than in serum and LIF (Figure S4). Therefore if *Nanog*^−/−^ embryos have the capacity to produce any epiblast cells, their recovery should be maximized in 2i/LIF. We harvested intercross blastocysts at E3.5 when the newly formed ICM is indistinguishable between mutant and wild-type. After culture in 2i or in 2i/LIF for 48 hr, all 43 Nanog-positive embryos had an ICM containing a large compartment of cells double positive for Oct4 and Nanog, with overlying Gata4-positive cells. In 10 Nanog-negative embryos the ICM had completely degenerated with neither Oct4 nor Gata4 detectable. Five of these displayed Eed foci in all cells (Figures 6A–6C). Lack of Gata4 staining in both freshly harvested and cultured Nanog-deficient blastocysts indicates that the loss of ICM does not arise by unscheduled differentiation into hypoblast, as we previously speculated (Chambers and Smith, 2004; Mitsui et al., 2003). Instead, these findings suggest that without Nanog, ICM cells are unable to progress into a correctly specified and viable epiblast and that hypoblast either does not form or rapidly degenerates.

### *Nanog*^−*/*−^ ICM Cells Can Differentiate into Trophoblast but Do Not Make Hypoblast

We investigated in more detail the fate and potency of *Nanog* null ICM cells. We stained intercross E4.5 embryos with Troma1 to detect cytokeratin 8 expressed by trophoblast (Nichols et al., 1998). We costained for Oct4 and activated caspase 3 to monitor apoptosis. Nanog immunostaining could not be used in this combination with available antibodies. Therefore mutant embryos were identified by low or absent Gata4. This correlated with reduced ICM cell numbers and increased caspase immunoreactivity (Figure 6D). In three out of four of these embryos some Oct4-positive cells costained withTroma1, which was never seen in the wild-type or heterozygous embryos at this stage (Figures 6E and 6F). These cells appeared to be on the surface of the embryo, suggesting incorporation into the trophoblast.

We isolated 56 ICMs from E3.5–E3.75 intercross blastocysts and cultured them in medium supplemented with LIF and serum. Individual genotypes were determined from the trophoblast lysates. Wild-type and heterozygous ICMs sustained a central cell mass and produced outgrowths of cells with refractile and migratory features characteristic of parietal endoderm (Figures 6G and 6H). In 3 out of 48 cases, outgrowths also contained large flattened cells of trophoblast morphology, suggesting that those ICMs had been harvested from early stage blastocysts and retained the capacity for trophoblast production (Nichols and Gardner, 1984). Eight out of eight null ICMs did not maintain any undifferentiated cell mass and either failed to attach and degenerated or produced a few trophoblast cells with spread morphology and large nuclei (Figure 6G). In no case did we observe either persistent ICM or hypoblast in a null embryo culture.

We conclude that Nanog mutant ICM cells are blocked in a transition stage of ICM development. Null cells cannot progress to pluripotency and therefore have only two options, differentiation into trophoblast or death.

## Discussion

**Figure 7:**
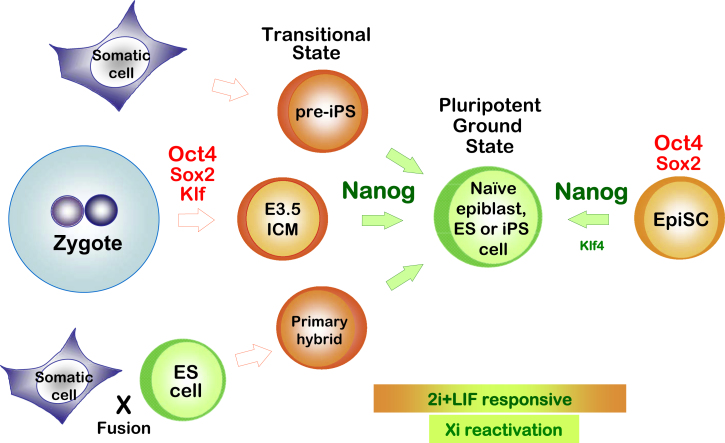
Nanog Orchestrates Synthesis of Ground State Pluripotency Acquisition of pluripotency proceeds via transitional states established by Oct4 with support from Sox2 and Klf4. Transitional cells have shed differentiated features and partially activated the pluripotent transcriptional circuitry. However, progression to the ground state requires the action of Nanog. Additionally in the case of iPS cells, reprogramming transgenes must be downregulated. In the case of EpiSCs, increased expression of Nanog can erase developmental priming and reinstate naive pluripotency, facilitated by Klf4. We propose that in each of these contexts Nanog directs synthesis of unstructured Oct4-, Sox2-, and Klf-mediated transcription into a coherent gene regulatory network, the pluripotent ground state.

Nanog can command constitutive self-renewal of ES cells (Chambers et al., 2003) and appears able to reverse precommitment perturbations of the pluripotent state (Chambers et al., 2007; Silva and Smith, 2008; Suzuki et al., 2006). Nanog also greatly increases the efficiency of nuclear reprogramming by ES cell fusion (Silva et al., 2006). It is therefore somewhat surprising that Nanog is not represented among the minimal combinations of exogenous factors found to convert mouse somatic cells into iPS cells (Takahashi and Yamanaka, 2006). This study offers an explanation (Figure 7). Our results indicate that Nanog is in fact decisive for attaining this pluripotent ground state. However, this requirement is during the final phase of reprogramming when other key factors are already present and may be fulfilled by activation of endogenous *Nanog*. A role in the culmination of somatic cell reprogramming is mirrored by the pivotal function revealed for Nanog in establishing pluripotency in the embryo.

Analyses of reprogramming in Nanog-deficient cells show that it is fully dispensable for the initial steps: loss of differentiated characteristics and creation of a pre-pluripotent state. Indeed pre-iPS cells, which have silenced somatic genes, exhibit expression of some markers of pluripotency but not Nanog (Silva et al., 2008; Sridharan et al., 2009). The failure to respond to 2i/LIF, which efficiently converts wild-type pre-iPS cells to pluripotency (Silva et al., 2008), indicates that *Nanog*^−/−^ pre-iPS cells cannot attain pluripotent status. This block is overcome, however, by introduction of Nanog. Therefore, Nanog mediates acquisition of pluripotency by dedifferentiated partially reprogrammed cells.

The in vivo phenotype of *Nanog* deletion shows that it is critical for early ICM cells to mature into pluripotent epiblast. This explains why mutant embryos cannot give rise to ES cells (Mitsui et al., 2003), even though Nanog-deficient ES cells are viable (Chambers et al., 2007). Cells that are allocated to the ICM but unable to upregulate Nanog progressively degenerate between E3.5 and E4.5. This occurs by a combination of differentiation into trophoblast and apoptosis (Figures 6D to 6F). The failure of Nanog null ICMs to form hypoblast is unexpected. It is possible that Nanog plays a role in potentiating hypoblast specification. Alternatively, hypoblast may be specified but unable to survive in the absence of paracrine support from a nascent epiblast. This would be consistent with the detection of occasional mutant cells showing weak Gata4 staining.

Oct4 and its partner Sox2 are expressed ubiquitously in morulae and early blastocysts and are present throughout the ICM until after segregation of the hypoblast (Avilion et al., 2003; Chazaud et al., 2006; Palmieri et al., 1994). Expression of these factors is therefore too wide to define the epiblast. Nanog, in contrast, is upregulated at the time of compaction. It may fluctuate in early ICM cells (Chazaud et al., 2006; Dietrich and Hiiragi, 2007) but subsequently is uniformly and exclusively expressed in the nascent epiblast. This restricted expression of Nanog is associated with and essential for prosecuting the transition to pluripotency in a subset of cells that already express Oct4 and Sox2. We propose that the presence of Nanog harnesses Oct4 and Sox2 to create ground state pluripotency. In this context, Nanog may be considered the specification factor for epiblast.

Erasure of X chromosome silencing is a unique epigenetic signature of arrival at authentic pluripotency. Presence of a silenced X chromosome in pre-pluripotent female embryo cells along with Oct4 and Sox2 mirrors the circumstances in partially reprogrammed pre-iPS cells. The timing of Nanog action in both somatic cell reprogramming and epiblast formation implies a requirement for coincident expression of other pluripotency factors. Previously, we showed that fusion of ES cells overexpressing Nanog with NS cells results in an equivalent pluripotent hybrid yield as fusion with ES cells, where there is no requirement for reprogramming (Silva et al., 2006). Thus, in the appropriate transcription factor context, Nanog levels can modulate reprogramming up to maximal efficiency. Moreover, stable Nanog overexpression in EpiSCs, which express most pluripotent factors, is sufficient to direct reprogramming upon withdrawal of the EpiSC maintainance factors Fgf and activin. Most strikingly, Nanog acts synergistically with the absent factor Klf4, such that transient expression of both is sufficient to convert EpiSCs to ground state pluripotency.

The reprogramming process can be divided into discrete stages related to the requirement for Nanog activity (Figure 7). The first stage culminates in the generation of dedifferentiated pre-pluripotent cells sustained by *Oct4*, *Sox2*, and *Klf4* transgenes. In some circumstances, influenced by factors such as character of the reprogramming vector and genetic background, cells are able to transit out of this stage stochastically and reach authentic pluripotency (Jaenisch and Young, 2008). In many cases, however, cells are blocked in the pre-iPS cell state (Okita et al., 2007; Silva et al., 2008). Cells in this condition express some markers of pluripotency but Nanog remains silenced (Silva et al., 2008; Sridharan et al., 2009). Transfer to serum-free 2i/LIF culture relieves the block and allows generation of pluripotency. Our findings establish that this culminating step is critically dependent on Nanog. How might Nanog achieve this? A recent comparison by chromatin immunoprecipitation of pre-iPS cells and fully reprogrammed iPS cells identified multiple genes that are co-occupied by Oct4, Sox2, and Klf4 in ES cells but not in pre-iPS cells (Sridharan et al., 2009). Many of these genes have been proposed as targets of Nanog in ES cells. Therefore, Nanog may orchestrate transition to the pluripotent ground state by facilitating cooperative binding of the core pluripotency factors to their cognate ES cell targets. Mechanisms could include Nanog-dependent recruitment of protein complexes comprised of the core factors or Nanog-dependent chromatin modulation to render loci accessible, as suggested for intron 1 of the *Xist* locus (Navarro and Avner, 2009).

In conclusion, our findings suggest that Nanog lies at the heart of a convergent mechanism for attaining the ground state of authentic pluripotency both in embryonic development and in the final phase of somatic cell reprogramming. We surmise that Nanog choreographs the emergent gene regulatory network of pluripotency into a functional and self-sustaining configuration. The next challenge is to elucidate precisely how Nanog brings about this synthesis.

## Experimental Procedures

*Nanog*^−/−^ NS cells were derived using the established NS cell derivation protocol (Conti et al., 2005) from E12.5 fetal forebrain of a chimeric fetus after flow cytometric purification of GFP-expressing null cells. *Nanog*^+/+^ NS cells were derived from E12.5 fetal forebrain of transgenic lines that constitutively express the fusion protein tauGFP and the puromycin resistance gene or that carry regulatory sequences of the mouse *Oct4* gene driving GFP and puromycin resistance (Silva et al., 2006). *Nanog*^−/−^ rescue NS cells were derived by transfection with pPyfloxNanogIPdsRedIB to obtain cells that express constitutively Nanog and puromycin resistance. *Nanog*^−/−^ pre-iPS cells were transfected using Nucleofection with 1 μg of PBfloxNanogPgkHygro plus 2–3 μg PBase expression vector, *pCAGPBase* (Guo et al., 2009), to obtain cells that express constitutively Nanog and Hygromycin resistance. EpiSCs derived from E5.5 Oct4GiP epiblast were cultured and transfected as described (Guo et al., 2009).

The *Nanog* null mutation generated by homologous recombination has been described previously (Mitsui et al., 2003). Genotypes of intercross blastocysts were inferred from presence or absence of Nanog immunostaining.

## Supplemental Data

Document S1. Supplemental Experimental Procedures, One Table, Ten Figures, and Supplemental References

Movie S1A. Immunostaining for Eed (Grey) and Oct4 (Green) Oct4 staining marks the ICM, while Eed staining alone represents trophectoderm nuclei. Note that a subset of ICM nuclei have undergone X_i_ reactivation whereas all trophectoderm nuclei display a X_i_.

Movie S1B. Immunostaining for Eed (Grey), Nanog (Red), and Gata4 (Blue) Note that only Nanog-stained nuclei exhibit reactivation of the X_i_. Movies S1A and S1B: X_i_ reactivation is exclusive to the nanog-positive cell population. Video shows reconstruction of confocal sections through an individual E4.5 female blastocyst.
