# Supplementary material for: Nanog Is the Gateway to the Pluripotent Ground State
Source: Cell. 2009 Aug 21;138(4):722–37. doi: 10.1016/j.cell.2009.07.039 (PMC3437554; doi:10.1016/j.cell.2009.07.039)
Supplement: Document S1. Supplemental Experimental Procedures, One Table, Ten Figures, and Supplemental References [file mmc1.pdf]

Cell, Volume *138*

Supplemental Data

Nanog Is the Gateway to the Pluripotent Ground State

Jose Silva, Jennifer Nichols, Thorold W. Theunissen, Ge Guo, Anouk L. van Oosten, Ornella Barrandon, Jason Wray, Shinya Yamanaka, Ian Chambers, and Austin Smith

Contents:

I. Supplemental Experimental Procedures
II. Supplemental Figure Legends
III. Supplemental Figures
IV. Supplemental References

I. Supplemental Experimental Procedures

Culture conditions

NS cells were maintained in serum free N2B27 (Stem Cell Sciences, catalogue number: SCS-SF-NB-02) or NDiff basal RHB-A (Stem Cell Sciences, catalogue number: SCS-SF-NB-01) supplemented with 10 ng/ml of both EGF and FGF-2. Plat-E cells (Morita et al., 2000) used to produce retroviruses were maintained in DMEM containing 10% FCS, 1 μg ml^{-1} puromycin and 10 μg ml^{-1} blasticidin S. ES cells and pre-iPS cells were cultured in GMEM containing 10% FCS, 1× NEAA, 1 mM sodium pyruvate, 0.1 mM 2-mercaptoethanol, supplemented with LIF (complete medium). STO fibroblasts treated with mitomycin-C were used as feeder layer for the expansion of pre-iPS cells. 2i-iPS cells were generated and maintained without feeders in N2B27 supplemented with LIF and 2i inhibitors (Bain et al., 2007; Ying et al., 2008), CHIR99021 (3μM) and

PD0325901 (1μM) obtained from the Division of Signal Transduction Therapy, University of Dundee.

Cell fusion

Mek inhibitor treatment of dsRed2 ES cells was performed using either PD184352 or PD0325901 at 3μM. For fusions, cells of each type (generally $1x10^7$) were combined in serum-free GMEM in a conical tube, pelleted, and supernatant aspirated. The pellet was broken by gentle tapping and 300 μl of 50% PEG 1500 (ROCHE) pre-warmed to 37°C were gently added. Cells were left in 50% PEG over a 3 min period with occasional stirring. Then 1 ml of medium was added over a period of 3 min. Subsequently, a further 3 ml of medium were added. Cells were spun down and supernatant discarded. The pellet was resuspended in complete ES cell medium and plated. Selection was applied 48 hours later for reactivation of the Oct4 reporter transgene using puromycin (1μg/ml). For fusions between NS cells constitutively expressing tauGFP and puromycin resistance and ES cells expressing dsRed2 and hygromycin resistance, primary fusion products were purified by flow cytometry 24 hours after PEG treatment using a Dako Cytomation MoFlo MLS sorter. An aliquot of the sorted population was analysed by flow cytometry to determine purity and the number of plated hybrids was calculated from these. Please note that sorting at this point precedes reprogramming (Do and Scholer, 2004; Silva et al., 2006; Tada et al., 2001). This strategy normalises for differences in cell fusion rate between different replicates representing an accurate quantification of somatic cell reprogramming by cell fusion. To eliminate non hybrids puromycin (1μg/ml) and hygromycin (250μg/ml) selection was applied on sorted cells 72hrs after plating. Selection for hybrids in fusions between Nanog null ES and Nanog null NS cells depended on constitutively expressing drug resistance genes, hygromycin (150μg/ml) and puromycin (1μg/ml) respectively. This selection strategy was inefficient due to cell confluence and to eliminate persisting unfused cells 2i+LIF medium supplemented with puromycin was used.

Retroviral infection and iPS cell induction

Retroviral infection was performed as described (Takahashi et al., 2007; Takahashi and Yamanaka, 2006) with minor modifications. Plat-E cells were seeded at 4 $\times$ 10^6 cells per 100-mm dish. The following day, 9 µg of pMX-based retroviral vectors for Oct4, Sox2, Klf4, or c-MycT58 were introduced separately into Plat-E cultures using 27 µl of FuGENE 6 transfection reagent. After 24 h, the medium was replaced with 10 ml of DMEM containing 10% FCS. Target cells (MEFs and NS cells) were seeded at 1.2 x 10^5 cells per 35-mm dish coated with gelatin. The following day, virus-containing supernatants from Plat-E cultures were filtered through a 0.45-µm cellulose acetate filter. Equal volumes of the supernatants were mixed and supplemented with polybrene at the final concentration of 4µg ml^{-1}. Cells were incubated in the virus/polybrene-containing supernatants for 24 h, then restored to NS cell culture medium. Three days after transduction, cultures were changed into ES cell medium. For further expansion pre-iPS cells were replated onto feeders at day 5 in medium containing serum and LIF. pMXs-gw plasmids; pMXs-Oct4, pMXs-Klf4 and pMXs-cMycT58 were obtained from Addgene repository.

Embryo collection

The *Nanog* null mutation generated by homologous recombination has been described previously (Mitsui et al., 2003). Genotypes of intercross blastocysts were inferred from presence or absence of Nanog immunostaining. For cultured ICMs genotypes were determined by PCR analysis of trophectoderm lysates (Nichols et al., 1998) using primers aatgggctgaccgcttcctcgtgctt, agtacctcagcctccagcagatgc and cagaatgcagacaggtctacagcccc. Diapause was induced by injecting pregnant female mice intraperitoneally with Tamoxifen (Sigma, T5648-1G; 10µg per mouse) and subcutaneously with Depo Provera (Pharmacia, MEDEP01); 3mg per mouse) at 2.5 days post coitum (dpc). Blastocysts were flushed from uteri four days later. Embryos were harvested at embryonic day (E) 3.5 or 3.75 and ICMs isolated by immunosurgery with collection of trophectoderm lysate for genotyping. ICMs were plated individually into gelatinised 4 well plates containing GMEM with 20% foetal calf serum and incubated for 8 days.

Immunoblotting

The following antibodies and dilutions were used: rabbit polyclonal antibody to Sox2 (1:2000) from Chemicon; mouse monoclonal antibodies to Oct4 (C-10) (1:500) and α-tubulin (1:5000 in W) from Santa Cruz Biotechnology and rabbit polyclonal antibody to Nanog (1:1000) from Abcam (ab21603-100).

Immunofluorescent staining

Blastocysts were fixed for 15 minutes in 4% PFA, rinsed in PBS containing 3mg/ml PVP, permeabilised in PBS/PVP with 0.25% triton X 100 for 30 minutes, and blocked in PBS containing 0.1% BSA, 0.01% Tween 20 and 2% donkey serum for 15 minutes, all at room temperature. Primary antibody solutions were made up in blocking buffer and embryos were incubated overnight at 4^oC. They were rinsed three times for 15 minutes each in blocking buffer then incubated for an hour at room temperature in the appropriate secondary antibodies at 1 in 500 in blocking buffer. After three rinses for 15 minutes each they were incubated briefly in increasing concentrations of Vectashield (with DAPI H-1200) then mounted in concentrated Vectashield and sealed on glass slides. Primary antibodies used were Oct4 (1:200) from Santa Cruz Biotechnology (C-10, sc-5279); Nanog (1:200) from Abcam (ab21603-100); activated Caspase 3 (1:100) from R&D Systems (AF-835); Gata4 (1:100) from Santa Cruz Biotechnology (C-20, sc-1237), Gata6 (1:100) from R&D Systems (AF1700), and Eed (1:10) gift from Arie Otte. Antibodies for NS cell staining were Nestin (1:10) from DSHB and Tuj1 (1:400) from Covance, and for pre-iPS cells were SSEA-1 (1:10) and Ecad (1:100) from DSHB. Alexa fluorescent secondary antibodies from Molecular Probes were used in all cases. Slides were analyzed by confocal microscopy (Leica TCS SP5) and processed with Leica software and Adobe Photoshop. Phase images of cells and blastocysts were collected using a Leica CTR microscope.

RT-PCR and qRT-PCR

For RT-PCR total RNA was extracted using the RNeasy kit (Qiagen), and cDNA generated using Superscript II (Invitrogen). For qRT-PCR total RNA was extracted using the RNeasy kit (Qiagen), and cDNA generated using Superscript III (Invitrogen). Relative expression levels of Fgf4, Nanog, Rex1, Oct4, Klf2, Klf4, Fgf5, Nr0b1, Lefty, Brachyury (T), Zeb2 and Snai2 were determined using the TaqMan Fast Universal PCR Master Mix (Applied Biosystems) and FAM-labeled TaqMan Gene Expression Assays. Relative expression levels of retroviral Oct4, Klf4, Sox2, c-Myc, and endogenous Oct4 and Sox2, were determined using Custom TaqMan Gene Expression Assays. Relative expression levels of Olig2 were determined using gene-specific primers and FAM-labeled probe no.21 from the Universal Probe Library (UPL) Set (Roche). Average threshold cycles were determined from triplicate reactions and the levels of gene expression were normalized to GAPDH (VIC-labeled endogenous control assay). Error bars indicate ±1 standard deviation, except in Fig. 2J and Fig. 3G were error bars indicate the range of fold change relative to the sample with highest expression. Relative expression levels of Xist and Fbx15 were determined using gene-specific primers and the Fast SYBR Green Master Mix (Applied Biosystems). Expression levels of Xist were normalized to β-actin, and calibrated relative to the NS -/- sample. Expression levels of Fbx15 were normalized to GAPDH (User Bulletin #2, Applied Biosystems, 2001). Error bars indicate ± 1 standard deviation. qRT-PCR experiments were performed on a 7900 HT Fast Real-Time PCR System or StepOnePlus Real Time PCR System (Applied Biosystems). See the Primer Table for details of primers and TaqMan gene expression assays used in this study.

Table S1. Primers used in this study

Gene	Primer sequence (5'- 3')	Application
Nanog-F	CAGGTGTTTGAGGGTAGCTC	RT-PCR
Nanog-R	CGGTTCATCATGGTACAGTC	
Oct4-F	GTGACTGCCTACCAGAATGA	RT-PCR
Oct4-R	ATTGTCCGCATAGGTTGGAG	
Rex1-F	TTGGGGCGAGCTCATTACTT	RT-PCR
Rex1-R	TTGCCACACTCTGCACACAC	
Fgf4-F	CACGAGGGACAGTCTTCTGG	RT-PCR
Fgf4-R	CGTCGGTAAAGAAAGGCACA	
Blbp-F	GGGTAAGACCCGAGTTCCTC	RT-PCR
Blbp-R	ATCACCACTTTGCCACCTTC	
Gapdh-F	CCCACTAACATCAAATGGGG	RT-PCR
Gapdh-R	CCTTCCACAATGCCAAAGTT	
Puromycin-F	CGGGTCACCGAGCTGCAAGAACTCTTC CTC	RT-PCR
Puromycin-R	CGCCAGGAGGCCTTCCATCTGTTGCTG	
Olig2-F	GGCGGTGGCTTCAAGTCATC	RT-PCR
Olig2-R	TAGTTTCGCGCCAGCAGCAG	
Olig2-F	AGACCGAGCCAACACCAG	UPL real time PCR
Olig2-R	AAGCTCTCGAATGATCCTTCTTT	
Xist-F	GGTTCTCTCTCCAGAAGCTAGGAAG	SYBR green real time PCR
Xist-R	TGGTAGATGGCATTGTGTATTATATGG	
Fbx15-F	TGTTAGAGGCTCATCTGTCACG	SYBR green real time PCR
Fbx15-R	GATGGCATTCTGTCCAGGGAT	
β-actin-F	CAACGAGCGGTTCCGATG	SYBR green real time PCR

β-actin-R	GCCACAGGATTCCATACCCA	
Gapdh-F	AGGTCGGTGTGAACGGATTTG	SYBR green real time PCR
Gapdh-R	TGTAGACCATGTAGTTGAGGTCA	
Retr. Oct4-F	TGGTACGGGAAATCACAAGTTTGTA	Custom TaqMan gene expression assay for retroviral transgene
Retr. Oct4-R	GGTGAGAAGGCGAAGTCTGAAG	
Retr. Oct4-probe	FAM-CACCTTCCCCATGGCTG-MGB	
Retr. Klf4-F	TGGTACGGGAAATCACAAGTTTGTA	Custom TaqMan gene expression assay for retroviral transgene
Retr. Klf4-R	GAGCAGAGCGTCGCTGA	
Retr. Klf4-probe	FAM-CCCCTTCACCATGGCTG-MGB	
Retr. cMyc-F	TGGTACGGGAAATCACAAGTTTGTA	Custom TaqMan gene expression assay for retroviral transgene
Retr. cMyc-R	GGTCATAGTTCCTGTTGGTGAAGTT	
Retr. Myc-probe	FAM-CCCTTCACCATGCCCC-MGB	
Retr. Sox2-F	TGGTACGGGAAATCACAAGTTTGTA	Custom TaqMan gene expression assay for retroviral transgene
Retr. Sox2-R	GCCCGGCGGCTTCA	
Retr. Sox2-probe	FAM-CTCCGTCTCCATCATGTTAT-MGB	
End. Oct4-F	TTCCACCAGGCCCCC	Custom TaqMan gene expression assay for endogenous transcript
End. Oct4-R	GGTGAGAAGGCGAAGTCTGAAG	
End. Oct4-probe	FAM-CCCACCTTCCCCATGGCT-MGB	
Endo. Sox2-F	CGGGCCGAGGGTTGG	Custom TaqMan gene expression assay for endogenous transcript
Endo. Sox2-R	CAGCTCCGTCTCCATCATGT	
Endo. Sox2-probe	FAM-CAGCGCCCGCATGTATA-MGB	
Fgf4	Applied Biosystems ID: Mm00438917_m1	TaqMan gene expression assay
Nanog	Applied Biosystems ID: Mm02384862_g1	TaqMan gene expression assay
Rex1	Applied Biosystems ID: Mm03053975_g1	TaqMan gene expression assay

Oct4 (total)	Applied Biosystems ID: Mm00658129_gH	TaqMan gene expression assay
Nr0b1	Applied Biosystems ID: Mm00431729_m1	TaqMan gene expression assay
Klf2	Applied Biosystems ID: Mm01244979_g1	TaqMan gene expression assay
Klf4	Applied Biosystems ID: Mm00516104_m1	TaqMan gene expression assay
Fgf5	Applied Biosystems ID: Mm00438919_m1	TaqMan gene expression assay
Lefty	Applied Biosystems ID: Mm00438615_m1	TaqMan gene expression assay
T (brachyury)	Applied Biosystems ID: Mm01318252_m1	TaqMan gene expression assay
Zeb2	Applied Biosystems ID: Mm00497193_m1	TaqMan gene expression assay
Snai2	Applied Biosystems ID: Mm00441531_m1	TaqMan gene expression assay
GAPDH	Applied Biosystems ID: 4352339E	TaqMan gene expression assay

II. Supplemental Figure Legends

Figure S1. Mek/Erk inhibition induces increased Nanog expression.
Immunostaining for Oct4 and Nanog in control and 3μM Mek inhibitor (PD0325901) treated ES cells cultured in serum and LIF. Identical settings were used to acquire the images for DAPI, Oct4 and Nanog for the Meki treated and untreated cells.

FigureS2. Gene expression analysis of ES^{Δ}xNS cell hybrid clones
RT-PCR analysis for Nanog, Oct4, Olig2 and blbp in ES^{Δ}xNS cell hybrids. RT-PCR analyses for puromycin (puro) resistance gene, Oct4, Olig2 and blbp in ES^{wt}xNS and $ES^{\Delta Ng}$xNS cell hybrids serve as a control.

Figure S3. Generation of *Nanog*$^{-/-}$ NS cells
(A) E12.5 chimeric foetus produced with *NanogΔ* ES cells.
(B) NanogΔ NS cells generated from chimeric embryo.
(C) Immunofluorescent staining of *NanogΔ* and *NanogΔ* + Ng NS cells for Nestin.
(D) Immunofluorescent staining of differentiated NanogΔ NS cells for neuronal marker Tuj1.

Figure S4. *Nanog*$^{-/-}$ ES cells self-renew in 2i culture conditions
(A) Phase contrast image of Nanog null ES cells cultured in 2i.
(B) Expression of undifferentiated ES cell markers by NanogΔ ES cells (RCNßH-B(t) and TßC44Cre6) (Chambers et al., 2007) cultured in 2i.

Figure S5. *Nanog* null pre-iPS cells express intermediate markers
(A) Wells containing Nanog null (Δ) NS, serum differentiated (diff) NS and pre-iPS cells, and Nanog rescued (Ng) Δ NS, serum diff NS and pre-iPS cells stained for Alkaline phosphatase activity (red).
(B) E-cadherin staining of partially reprogrammed cells.
(C) Immunostaining for SSEA1 of *NanogΔ* pre-iPS cells.

Figure S6. Nanog is not required for iPS cell self-renewal.

(A) NanogΔ iPS cell colony following Cre deletion of Nanog transgene.

(B) Quantitative gene expression analysis for Nanog, Fgf4 and Rex1 in Δ+Ng and Δ+Ng Cre iPS cells grown in 2i /LIF (L) or in Serum(S) L medium. Error bars indicate ±1 standard deviation.

(C) Quantitative gene expression analysis for Xist in Δ+Ng cre iPS cells grown in 2iL or SL medium. Analysis of parental wt and *Nanog*Δ ES cells serve as controls. Error bars indicate ±1 standard deviation.

(D) E12.5 chimeric foetus generated from Δ+Ng iPS cells after Cre deletion of the *Nanog* transgene.

Figure S7. Embryo-fibroblast derived clonal line of pre-iPS cells expresses transitional marker profile and is efficiently converted to authentic iPS cell status by 2i/LIF

(A) Quantitative gene expression analysis for fibroblast markers Zeb2 and Snai2, for retroviral (retro) Oct4, retro Klf4, retro cMyc, retro Sox2, and for pluripotency markers Fgf4, Fbx15, endogenous (endo) Oct4, endo Sox2, Nanog and Rex1 in MEFs and derivative pre-iPS and iPS cells. Error bars indicate ±1 standard deviation.

(B) Immunostaining for trimethylH3K27 (me3H3K27) and Oct4 in Mef pre-iPS cells.

(C) Flow cytometry dot plots of Oct4-GFP expression during pre-iPS to iPS cell conversion in 2i/LIF. These data were used to derive histogram in Figure 2J.

(D) Chimera generated from MEF pre-iPS cell clone derived iPS cells with C57BL/6 mate and pups. The recipient blastocyst strain was C57BL/6 so agouti offspring demonstrates transmission of the iPS cell haploid genome.

Figure S8. iPS cells generated from EpiSCs by transient expression of Nanog plus Klf4 can efficiently colonise the developing embryo

Without reprogramming, EpiSCs are completely unable to colonise the developing embryo after either morula aggregation (Guo et al., 2009) or blastocyst injection (Tesar

et al., 2007). Cells from two Epi-iPS cell clones were combined with E2.5 morulae. Aggregates were maintained in culture for 48 hours then transferred to pseudopregnant recipients. Embryos were harvested at E6.5 and examined by fluorescence microscopy for Oct4-GFP reporter expression. Images show two representative embryos with GFP expression throughout the egg cylinder demonstrating incorporation of Epi-iPS cells.

Figure S9. Nanog negative blastocysts express Oct4 throughout the ICM at E3. 5
(A) Nanog and Oct4 immunostaining of E3.5 blastocysts from intercrosses of *Nanog*$^{+/-}$ mice.
(B) Phase images of E3.5 blastocysts from intercrosses of *Nanog*$^{+/-}$ mice. Nanog negative embryos are presumed nulls.

Figure S10 Loss of Nanog expression does not acutely trigger X inactivation. Immunostaining for Nanog, Oct4 and Eed of XX ES cells cultured in 2i/LIF+N2B27 (0hrs) or in N2B27 alone for 48 and 96hrs.

III. Supplemental Figures

Figure S1

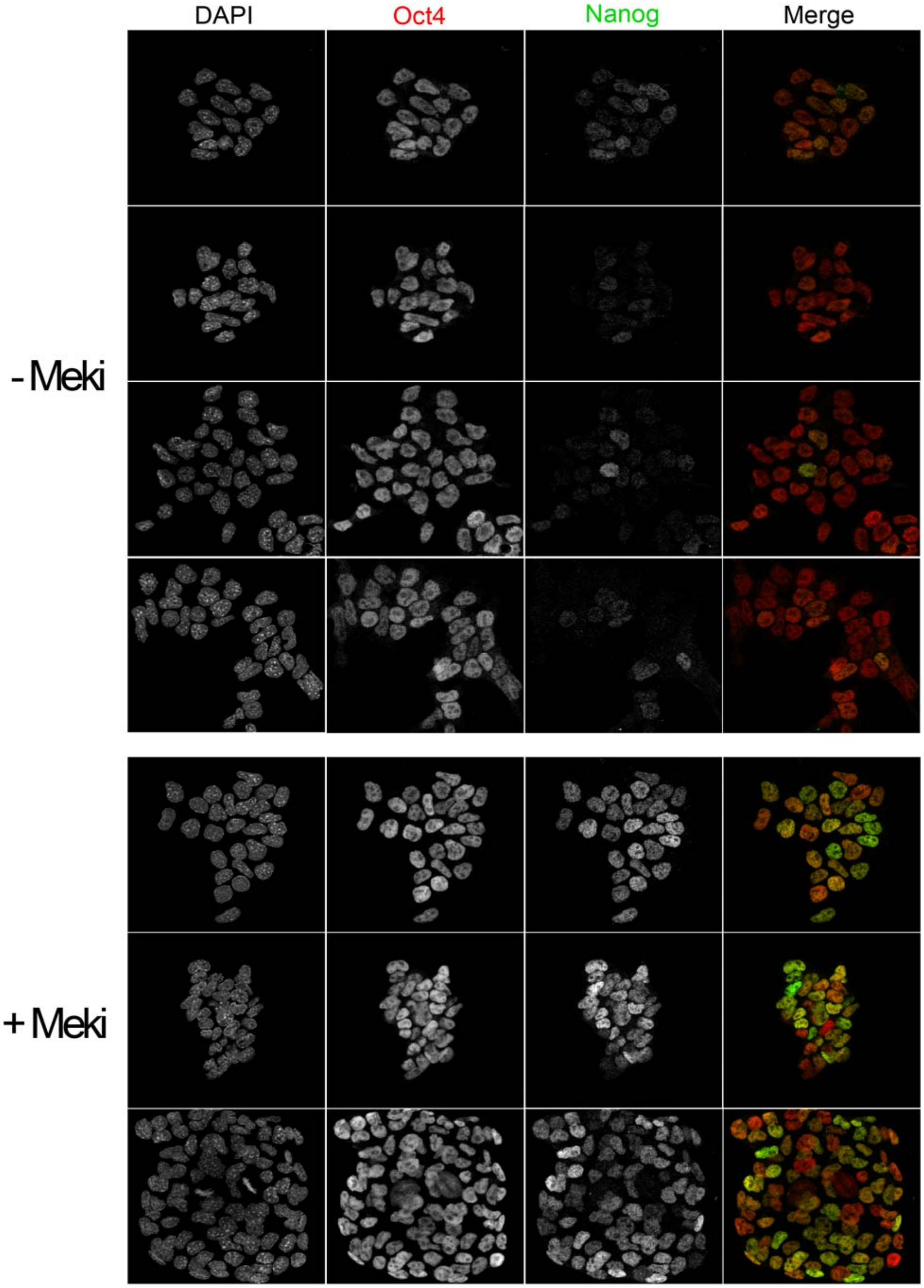

Figure S2

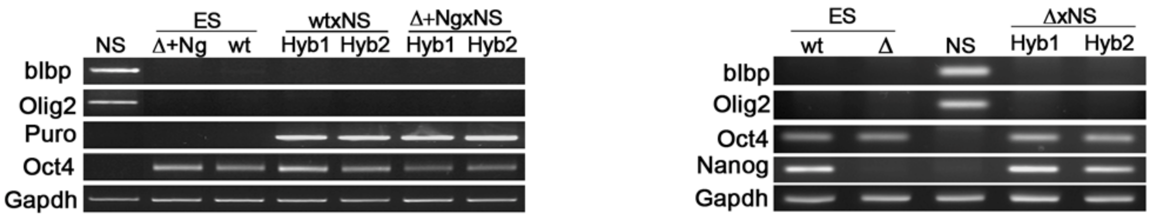
ES
wtxNS
Δ+NgxNS
NS
Δ+Ng
wt
Hyb1
Hyb2
Hyb1
Hyb2
blbp
Olig2
Puro
Oct4
Gapdh
ES
ΔxNS
wt
Δ
NS
Hyb1
Hyb2
blbp
Olig2
Oct4
Nanog
Gapdh

Figure S3

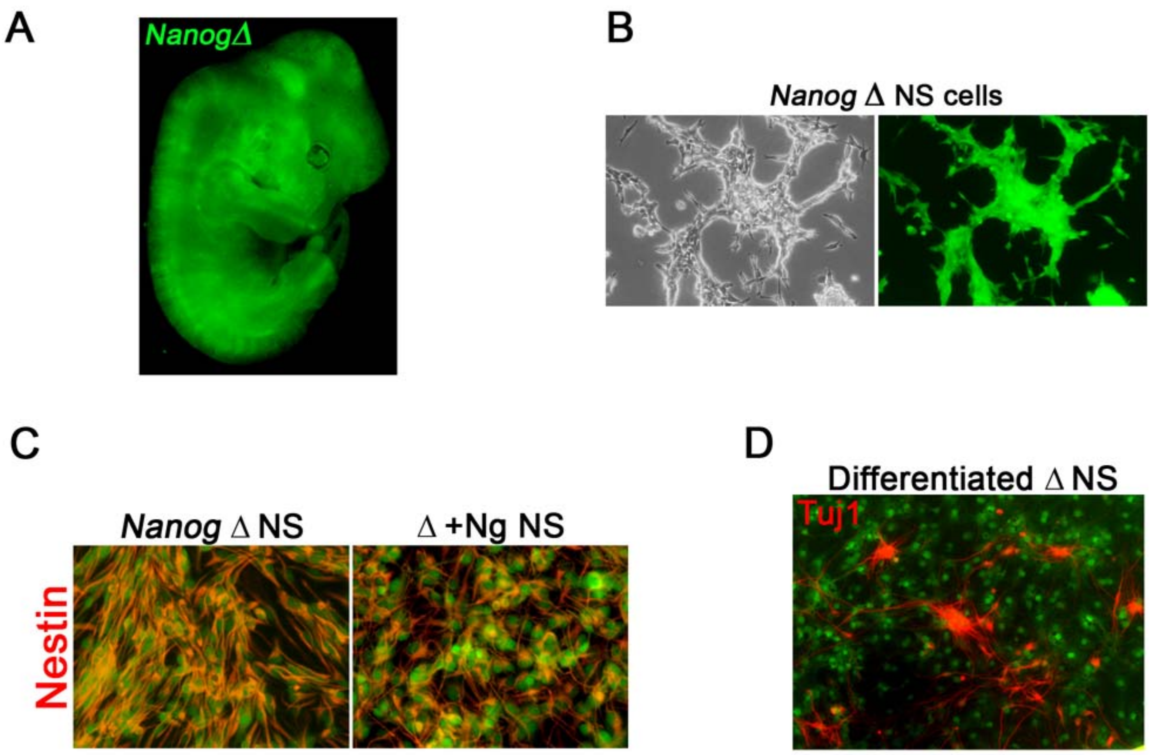
A
NanogΔ
B
Nanog Δ NS cells
C
Nanog Δ NS
Δ +Ng NS
Nestin
D
Differentiated Δ NS
Tuj1

Figure S4

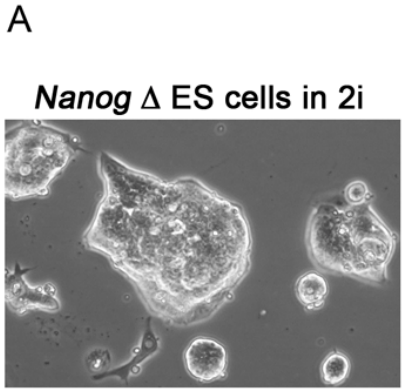
A
Nanog Δ ES cells in 2i

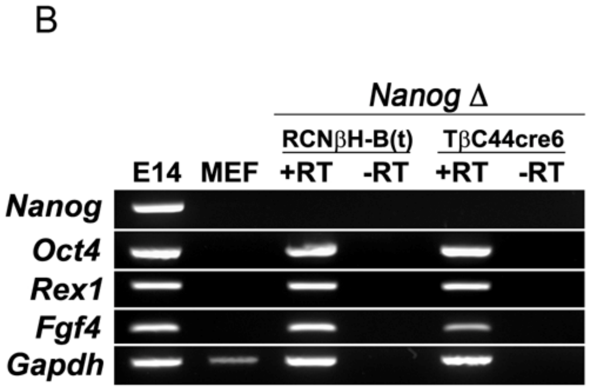
B
Nanog Δ
RCNβH-B(t)
TβC44cre6
E14
MEF
+RT
-RT
+RT
-RT
Nanog
Oct4
Rex1
Fgf4
Gapdh

Figure S5

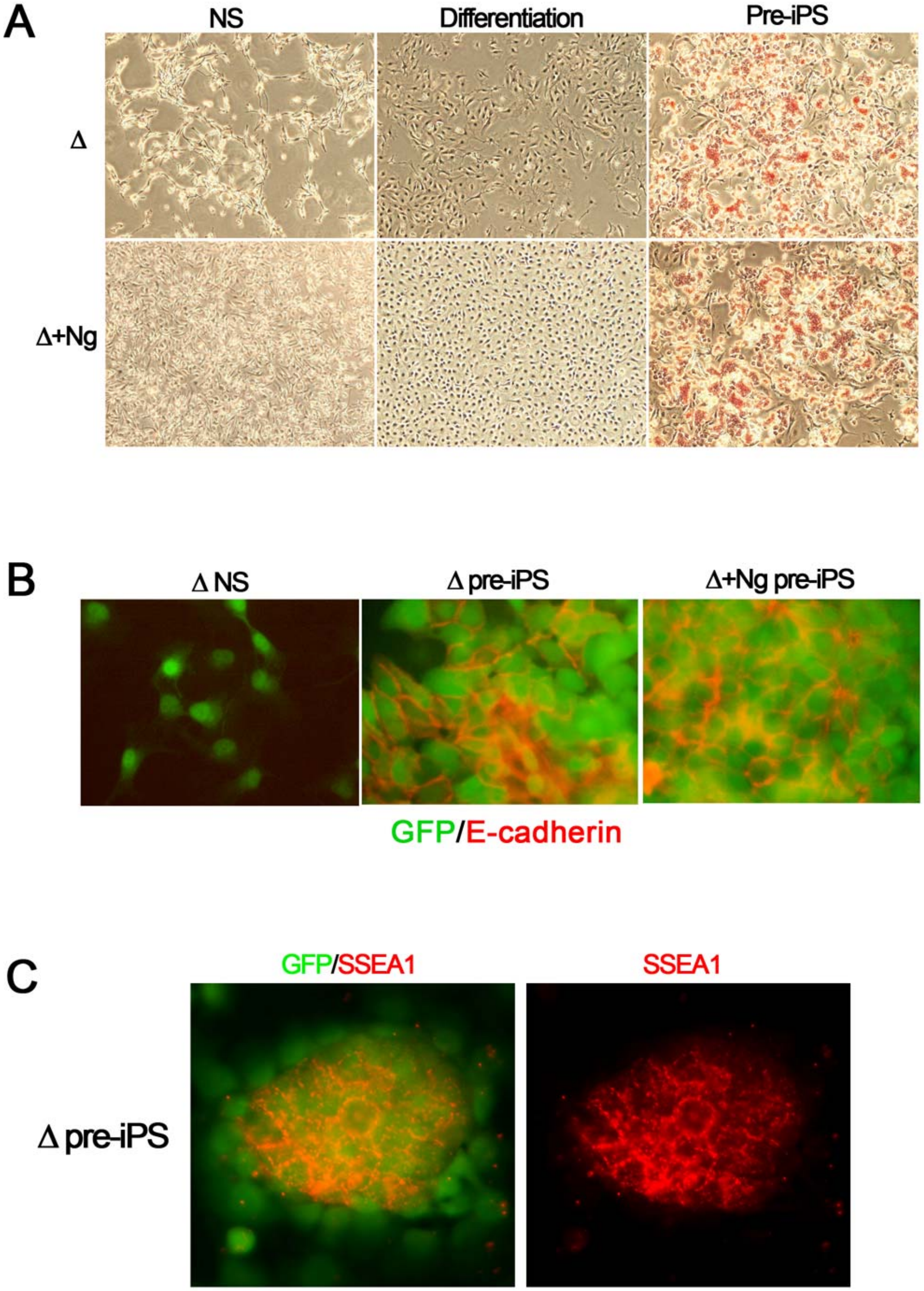
A
NS
Differentiation
Pre-iPS
Δ
Δ+Ng
B
Δ NS
Δ pre-iPS
Δ+Ng pre-iPS
GFP/E-cadherin
C
GFP/SSEA1
SSEA1
Δ pre-iPS

Figure S6

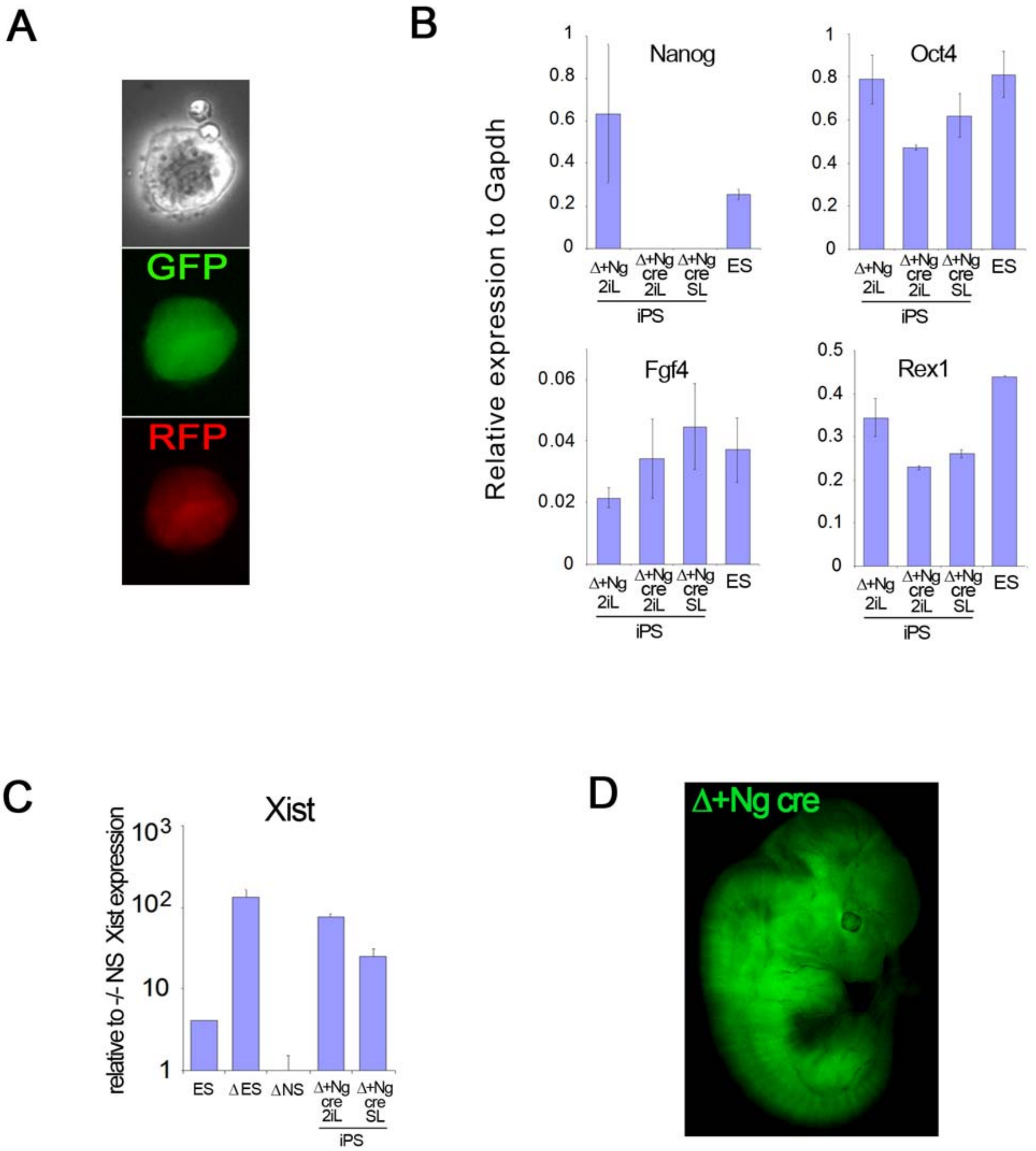
A
GFP
RFP
B
Relative expression to Gapdh
Nanog
Oct4
Fgf4
Rex1
Δ+Ng 2iL
Δ+Ng cre 2iL
Δ+Ng cre SL
ES
iPS
C
Xist
relative to -/- NS Xist expression
ES
ΔES
ΔNS
Δ+Ng cre 2iL
Δ+Ng cre SL
iPS
D
Δ+Ng cre

Figure S7

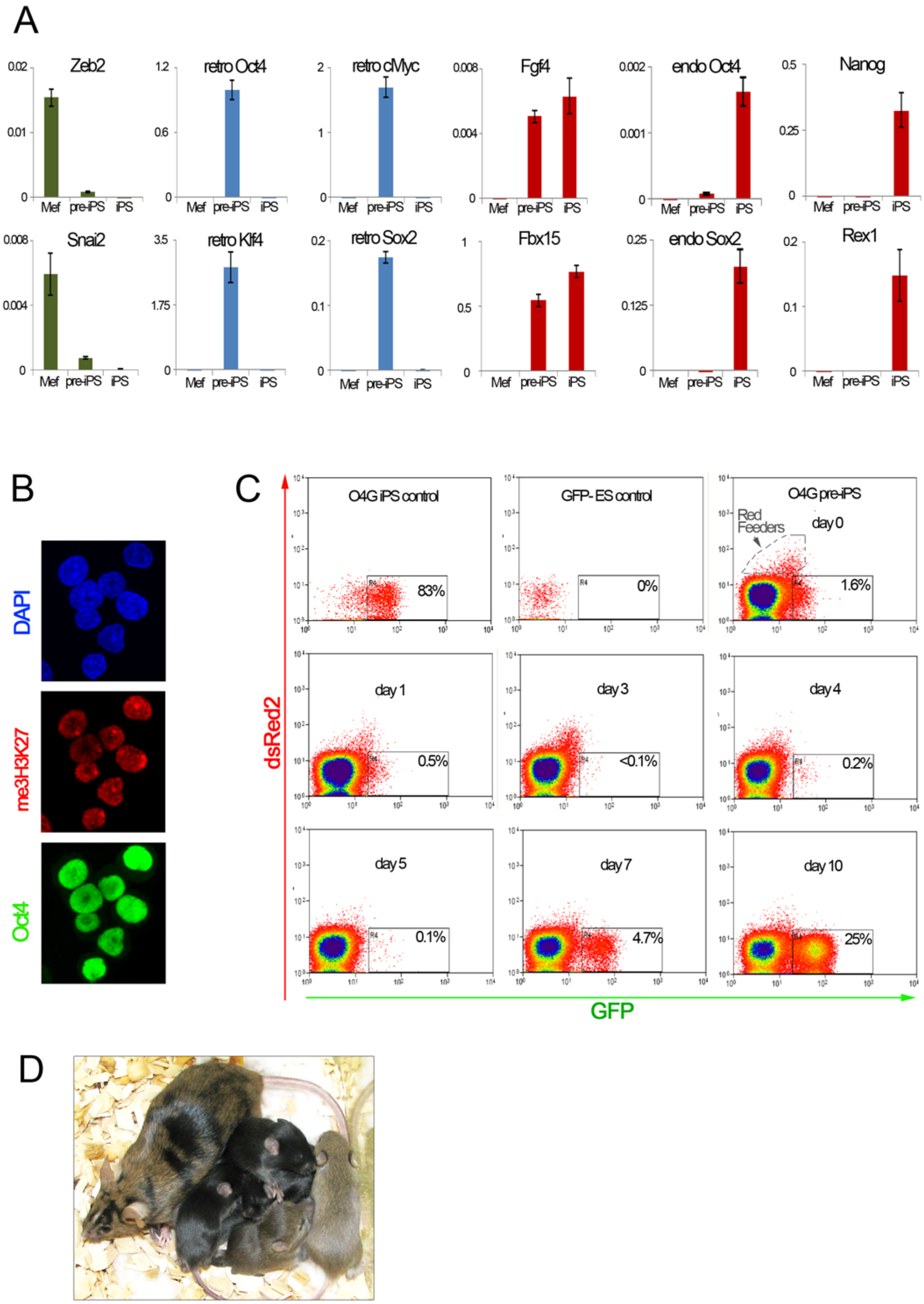
A
Zeb2
retro Oct4
retro cMyc
Fgf4
endo Oct4
Nanog
Snai2
retro Klf4
retro Sox2
Fbx15
endo Sox2
Rex1
Mef
pre-iPS
iPS
B
DAPI
me3H3K27
Oct4
C
O4G iPS control
GFP- ES control
O4G pre-iPS
Red Feeders
day 0
83%
0%
1.6%
day 1
day 3
day 4
0.5%
<0.1%
0.2%
day 5
day 7
day 10
0.1%
4.7%
25%
dsRed2
GFP
D

Figure S8

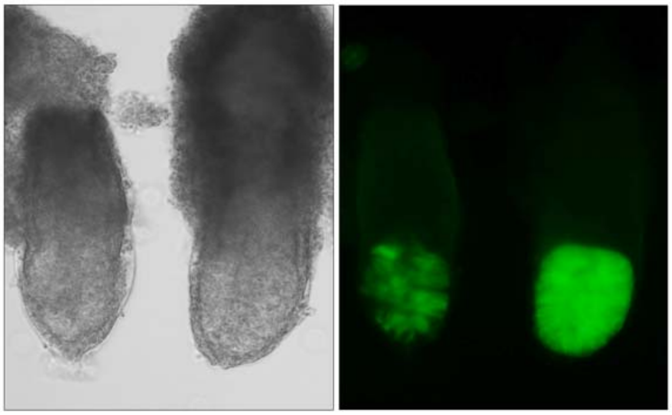

	Transient Nanog-Klf4 Epi-iPS	
	clone 1	clone 2
Chimeras / Total	22/32	16/18

Figure S9

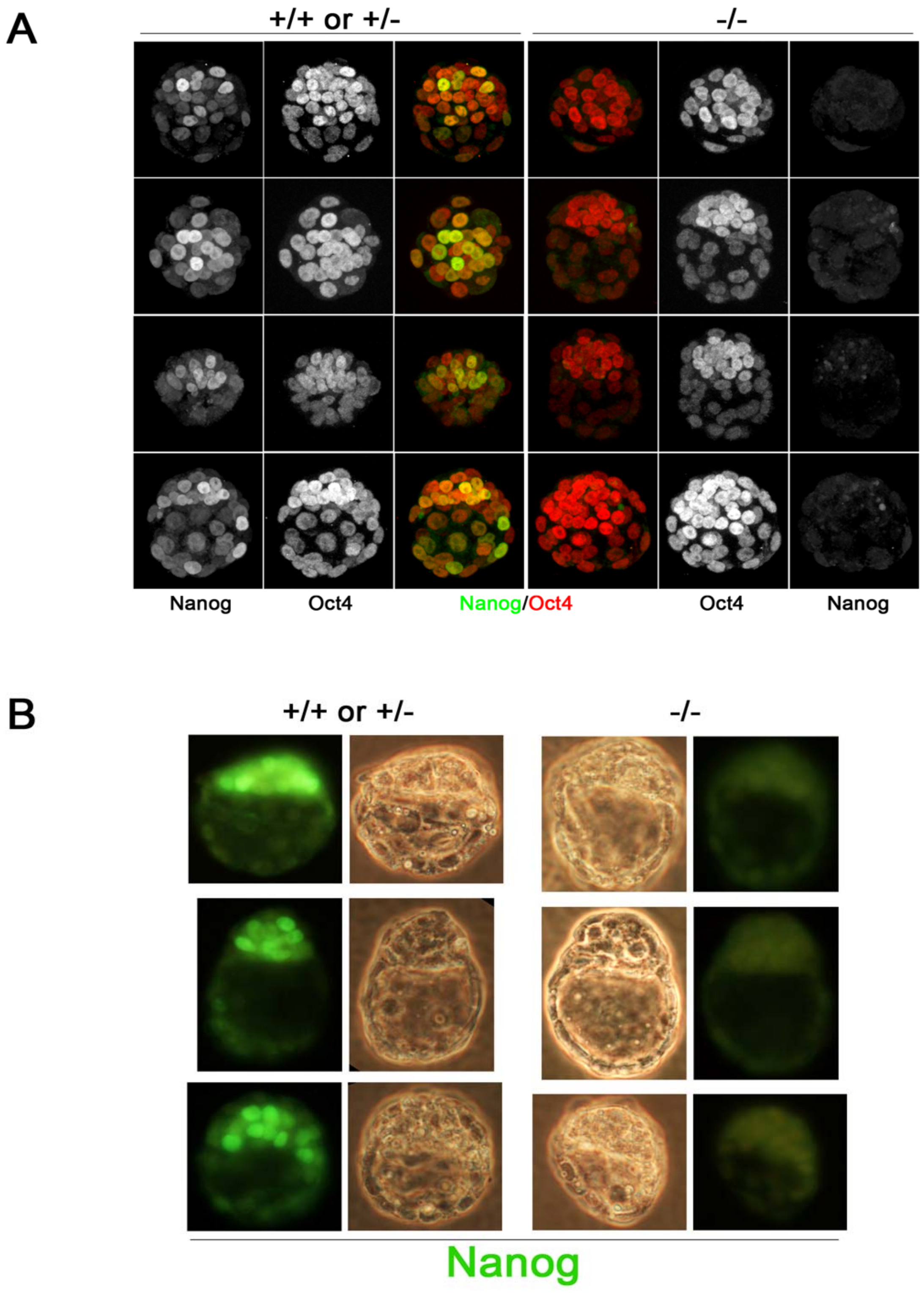

Figure S10

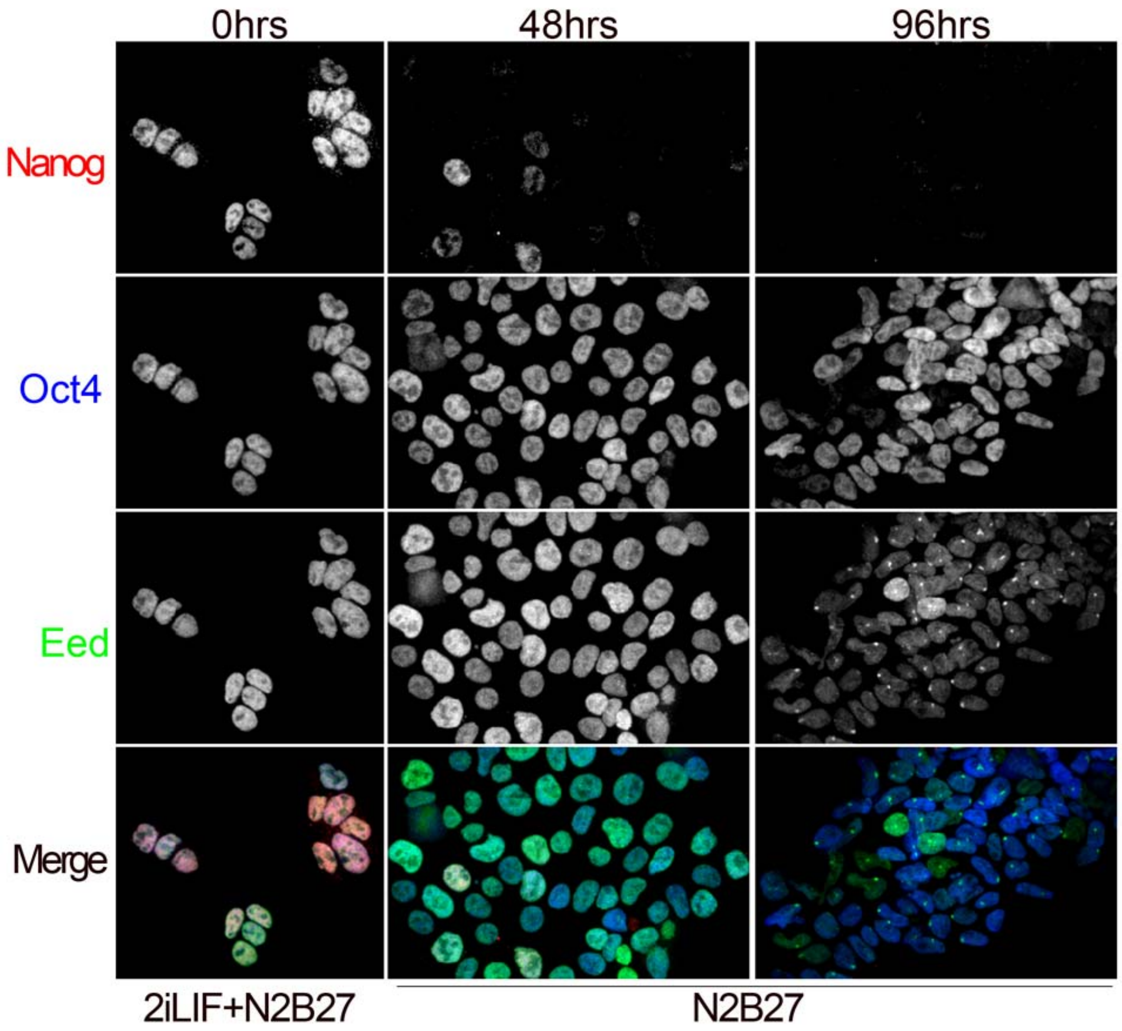

IV. Supplemental References

Bain, J., Plater, L., Elliott, M., Shpiro, N., Hastie, C.J., McLauchlan, H., Klevernic, I., Arthur, J.S., Alessi, D.R., and Cohen, P. (2007). The selectivity of protein kinase inhibitors: a further update. Biochem J *408*, 297-315.

Chambers, I., Silva, J., Colby, D., Nichols, J., Nijmeijer, B., Robertson, M., Vrana, J., Jones, K., Grotewold, L., and Smith, A. (2007). Nanog safeguards pluripotency and mediates germline development. Nature *450*, 1230-1234.

Do, J.T., and Scholer, H.R. (2004). Nuclei of embryonic stem cells reprogram somatic cells. Stem Cells *22*, 941-949.

Guo, G., Yang, J., Nichols, J., Hall, J.S., Eyres, I., Mansfield, W., and Smith, A. (2009). Klf4 reverts developmentally programmed restriction of ground state pluripotency. Development *136*, 1063-1069.

Mitsui, K., Tokuzawa, Y., Itoh, H., Segawa, K., Murakami, M., Takahashi, K., Marauyama, M., Maeda, M., and Yamanaka, S. (2003). The homeoprotein Nanog is required for maintenance of pluripotency in mouse epiblast and ES cells. Cell *113*, 631-642.

Morita, S., Kojima, T., and Kitamura, T. (2000). Plat-E: an efficient and stable system for transient packaging of retroviruses. Gene Ther *7*, 1063-1066.

Nichols, J., Zevnik, B., Anastassiadis, K., Niwa, H., Klewe-Nebenius, D., Chambers, I., Scholer, H., and Smith, A. (1998). Formation of pluripotent stem cells in the mammalian embryo depends on the POU transcription factor Oct4. Cell *95*, 379-391.

Silva, J., Chambers, I., Pollard, S., and Smith, A. (2006). Nanog promotes transfer of pluripotency after cell fusion. Nature *441*, 997-1001.

Tada, M., Takahama, Y., Abe, K., Nakatsuji, N., and Tada, T. (2001). Nuclear reprogramming of somatic cells by in vitro hybridization with ES cells. Curr Biol *11*, 1553-1558.

Takahashi, K., Okita, K., Nakagawa, M., and Yamanaka, S. (2007). Induction of pluripotent stem cells from fibroblast cultures. Nat Protoc *2*, 3081-3089.

Takahashi, K., and Yamanaka, S. (2006). Induction of pluripotent stem cells from mouse embryonic and adult fibroblast cultures by defined factors. Cell *126*, 663-676.

Tesar, P.J., Chenoweth, J.G., Brook, F.A., Davies, T.J., Evans, E.P., Mack, D.L., Gardner, R.L., and McKay, R.D. (2007). New cell lines from mouse epiblast share defining features with human embryonic stem cells. Nature *448*, 196-199.

Ying, Q.L., Wray, J., Nichols, J., Batlle-Morera, L., Doble, B., Woodgett, J., Cohen, P., and Smith, A. (2008). The ground state of embryonic stem cell self-renewal. Nature *453*, 519-523.
